# Life-skills training program: its effect on self-efficacy among patients with substance use disorders

**DOI:** 10.1186/s40359-025-03907-2

**Published:** 2026-01-21

**Authors:** Abelmouttelb Abdelkawy Abdelmouttelb, Sahar Mahmoud Mohamed Elewa, Fatma Ata Abdelsalhen, Fatma Mohammed Ibrahim

**Affiliations:** Psychiatric Mental Health Nursing Department, Faculty of Nursing, Ain Shams University, Cairo, Egypt

**Keywords:** Life-Skills, Substance use disorders, Self-efficacy, Relapse

## Abstract

**Background:**

Substance Use Disorders (SUDs) affect 5–6% of the global population, leading to substantial psychological and social burdens. Enhancing self-efficacy is central to recovery and relapse prevention, and life-skills training programs show promise in this regard. Yet, evidence from low-resource settings remains scarce.

**Aim:**

This study aimed to evaluate the effect of life-skills training program on self-efficacy among patients with substance use disorders.

**Method:**

A quasi-experimental one-group pretest-post-test design was used. A purposive sample of 45 male patients with Substance Use Disorders admitted in male addiction treatment and rehabilitation building at El Abbasia Mental Health Hospital )affiliated with the General Secretariat of Mental Health and Addiction Treatment (GSMHAT), Ministry of Health and Population, Cairo, Egypt( received a structured life-skills training program consisting of 15 group-sessions delivered twice weekly, focusing on behavioral, emotional, and wellness skills to enhance self-efficacy and prevent relapse. Self-efficacy was measured before and after the intervention using the Arabic version of the Alcohol Abstinence Self-Efficacy Scale (AASE). Data was analyzed using paired sample t-tests. Results: Patients showed a statistically significant improvement in overall self-efficacy scores following the intervention (t= -50.27, *p* < 0.001), with notable improvement across all subscales including negative affect, social pressure, physical discomfort, and cravings.

**Conclusions:**

Life skills training program had a positive effect on self-efficacy among patients with substance use disorders. Therefore, integrating life-skills training into rehabilitation and relapse prevention programs for patients with substance use disorders to enhance their capabilities and strengthen their self-efficacy.

**Trial registration:**

ClinicalTrials.gov, NCT07174960. Retrospectively registered on 12 September 2025.

**Supplementary Information:**

The online version contains supplementary material available at 10.1186/s40359-025-03907-2.

## Introduction

Substance use disorders (SUDs) represent a spectrum of problematic substance use that includes impaired control, social impairment (for example, failure to meet major responsibilities), risky use (continued use despite clear dangers), and pharmacological features (tolerance and withdrawal) [56]. According to *the Diagnostic and Statistical Manual of Mental Disorders* (DSM 5), SUDs are defined by symptoms of tolerance, withdrawal, and compulsive use despite harmful consequences [57]. In addition, impaired control is a core feature of substance use disorders, marked by compulsive substance use that exceeds intended limits. It reflects disrupted executive regulation, where cravings overpower efforts to stop, making it a major factor contributing to relapse [61, 64].

The global burden of SUDs is significant. Reports from the World Health Organization (WHO) and the United Nations Office on Drugs and Crime (UNODC) show that about 5.5% of adults’ population were affected by SUDs in 2023 [62]. In the Middle East and North Africa (MENA) region, cases increased by 128.1% between 1990 and 2019, with a prevalence rate of 4.1% [45]. In Egypt, the prevalence of SUDs is estimated at 5.9%. Young people are the most exposed to substance abuse, while those aged 25–35 years most frequently seek treatment The disorder is more common in men, with prevalence among women remaining very low, which reflects a strong gender difference [3]. One-month prevalence ranges between 5.4% and 11.5%, and hospital-based data show an 11.2% incidence of SUDs among emergency cases [5]. National surveys in Egypt report a lifetime prevalence of substance use ranging from 7.25% to 14.5%, with SUDs affecting around 1.6% of the population [23].

A major challenge in SUD treatment is relapse. The Substance Abuse and Mental Health Services Administration (SAMHSA) defines relapse as a return to substance use after a period of abstinence or improvement [51]. Relapse is not viewed as failure but as a common part of the recovery process, occurring as either a single episode or a return to regular use [41, 58]. Evidence shows relapse rates can reach 40–75% within three weeks to six months after treatment [43]. Early relapse (short-term abstinence) is often linked to depression, unemployment, and weak social support, while late relapse (after long abstinence) is more related to poor coping skills, reduced self-efficacy, and lack of insight into substance use problems [42, 48].

Self-efficacy theory explains how personal beliefs shape behavior. Self-efficacy is the individual’s belief in their ability to perform the actions needed to reach goals and overcome challenges [8]. When treatment raises realistic expectations and confidence, clients are more likely to adopt healthy behaviors and avoid harmful ones [33]. Self-efficacy is not only about ability but also about believing one has the skills to manage difficult conditions [9]. People with higher self-efficacy act more actively to take control of their lives. In contrast, low self-efficacy is associated with shame, helplessness, and increased risk of relapse [7, 38].

In the context of SUDs, abstinence self-efficacy (ASE) refers to confidence in avoiding drug use over time [47]. Research shows that higher self-efficacy predicts better outcomes in both short- and long-term remission [15]. Skills-training activities during treatment significantly improve self-efficacy, which in turn reduces relapse risk as higher self-efficacy is associated with better treatment outcomes [20, 28, 40]. Life-skills training helps strengthen confidence in managing life, which increases resilience and reduces the need to use substances when facing challenges [7, 59, 63].

Life-skills training has been shown to improve functioning, reduce withdrawal symptoms, increase coping ability, and lower relapse risk [1, 32, 37, 38]. Patients with SUDs often lack supportive relationships and problem-solving abilities, which lowers their self-efficacy. Training in social and emotional skills such as communication, decision-making, critical thinking, and stress management helps individuals build stronger connections and handle life more effectively [44, 60]. Programs that teach coping strategies, anger control, and resistance to peer pressure also reduce relapse risk [27, 46].

Evidence supports the role of life-skills programs in improving self-efficacy. For example [49], found that stress management and coping skills training significantly increased self-efficacy in treatment settings, leading to better abstinence outcomes. Structured practice, homework focused on high-risk situations, and support from programs like the 12-step model provide mastery experiences that further strengthen self-efficacy.

Therefore, the current study aims to assess the impression of life-skills training program on self-efficacy among patients with substance use disorders.

## Aim of the study

Based on this background, the current study aims to evaluate the effect of a life-skills training program on self-efficacy among patients with substance use disorders after ten-week group intervention.

### Research hypothesis

#### H₁

Patients with substance use disorders who receive the life-skills training program will show a statistically significant improvement in their self-efficacy scores (as measured by the Alcohol Abstinence Self-Efficacy Scale) after a ten-week group intervention compared to their pre-intervention scores.

## Subjects & methods

### Study design

A quasi-experimental one-group pretest-post-test design was used to conduct the current study.

### Study setting

The study was conducted in male addiction treatment and rehabilitation building at El Abbasia Mental Health Hospital, affiliated with the General Secretariat of Mental Health and Addiction Treatment (GSMHAT), Ministry of Health and Population, Cairo, Egypt. The setting included detoxification (approximately 30 beds) and rehabilitation wards (60 beds), distributed across the second to fourth floors.

### Subjects

The study sample included hospitalized adult male patients diagnosed with substance use disorders, including those with alcohol use disorder and individuals currently using tobacco products (both conventional cigarette smokers and waterpipe/shisha users), all of whom had experienced at least one previous relapse episode. All participants had completed a minimum of three weeks in detoxification and were actively involved in a structured rehabilitation program at the time of the study. A purposive sampling technique was used to recruit eligible participants. Patients with chronic physical illnesses (e.g., diabetes, hypertension, or viral hepatitis) or comorbid psychiatric disorders (e.g., schizophrenia, bipolar disorder, or depression) were excluded. Approval was granted to access medical records, and all participants’ diagnoses were confirmed by qualified psychiatrists to ensure adherence to the inclusion criteria.

### Sample size

The required sample size was calculated using G*Power software version 3.1.9.7 [21] to conduct a two-tailed paired-samples t-test. Assuming a medium effect size (dz = 0.5), a significance level (α) of 0.05, and a statistical power (1–β) of 0.85, which is slightly higher than the conventional 0.80, to reduce the likelihood of Type II error and to increase the sensitivity of the study in detecting the effect of the intervention on self-efficacy, the analysis indicated that a total sample of 38 participants would be sufficient to detect a statistically significant difference between the pre- and post-intervention scores. However, since no control group was included in this study, the sample size was deliberately increased to 45 to strengthen the robustness of the analysis, minimize the impact of potential dropout or missing data, and ensure adequate statistical power despite the absence of a comparator group.

Participant recruitment and selection followed a multi-stage process. Initially, 75 patients were invited to participate. Of these, 66 patients provided consent and were enrolled. Subsequently, eligibility screening against predefined inclusion and exclusion criteria was conducted, resulting in 55 eligible participants. During the pre-test assessment phase, seven participants were excluded due to incomplete questionnaire responses. Prior to the commencement of the intervention program, a further three participants were discharged from care and consequently excluded. Therefore, the final analytical sample comprised 45 participants. See more fig in (1).

**Fig. 1:**
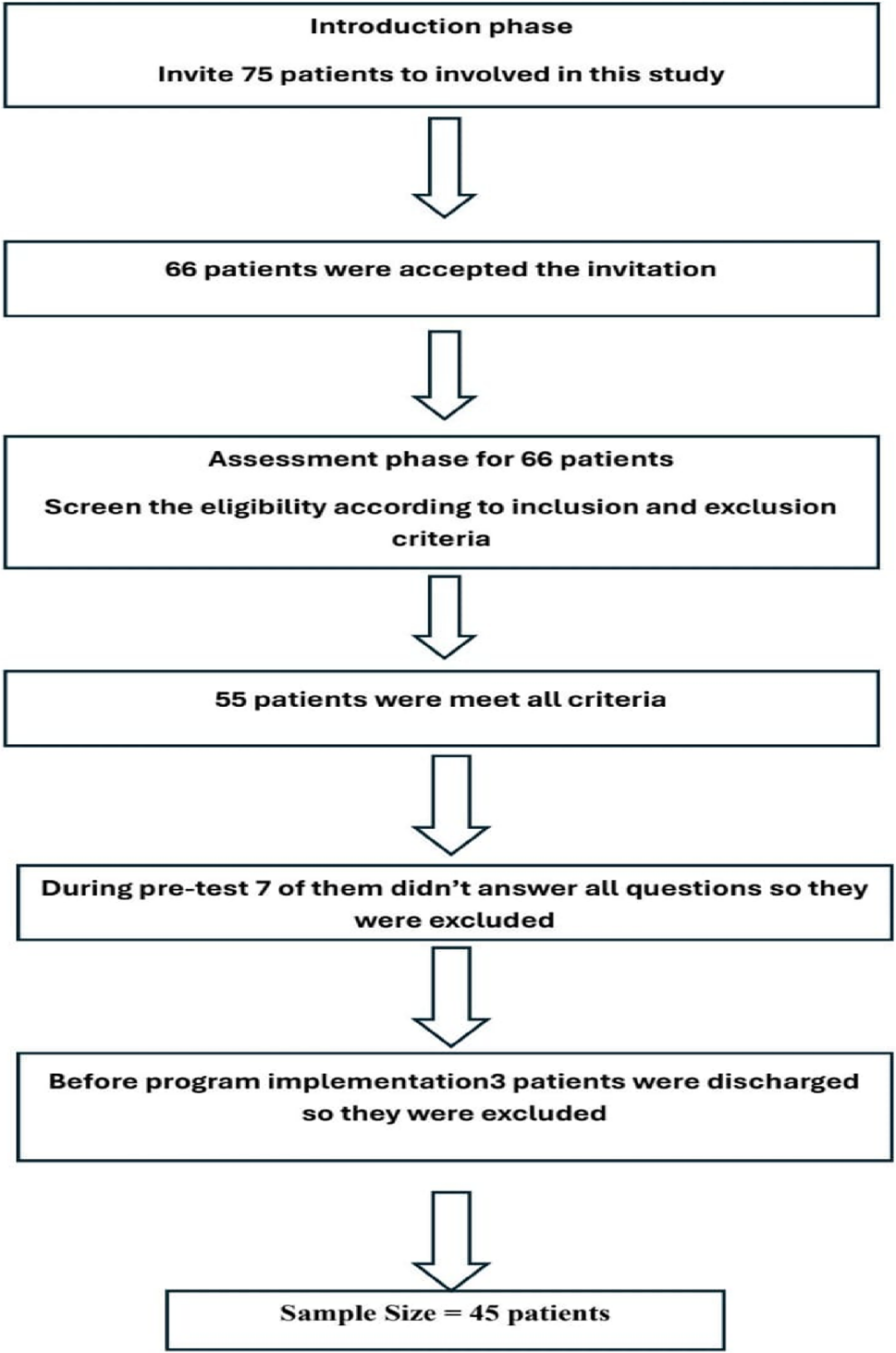
Flowchart of Sample Recruitment

### Tools of data collection

#### Tool I: interview questionnaire

##### Patients’ characteristics

Included age, marital status, educational level, occupation, place of residence, monthly income and number of family members.

##### Clinical Data (Addiction History)

Covered detailed information regarding the substance use history, including the types of substances used, age of onset, duration of use, route of administration, history of previous treatment attempts, types of treatment admissions, and presence of substance cravings. 

#### Tool II: Self-Efficacy scale (Adapted AASE)

Self-efficacy was measured using the Alcohol Abstinence Self-Efficacy Scale (AASE) [17], a validated 40-item questionnaire that assesses confidence and temptation in four high-risk situations: negative affect (10 statements), social interactions and positive states (10 statements), physical and other concerns (10 statements), and withdrawal/urges (10 statements). In this study, the word “alcohol” was replaced with “substance use” while keeping the original structure and scoring system. The linguistic modification didn’t alter the original meaning structure or purpose of the scale as the construction of self-efficacy to resist substance use parallels that of alcohol abstinence self-efficacy.

The scale was translated into Arabic using a forward–backward translation method. Two bilingual experts translated the items into Arabic, and two different bilingual translators, who did not see the original, translated them back into English. A panel of five experts (two professors of psychiatry, two professors of psychiatric/mental health nursing, and one clinical psychologist) reviewed the items and confirmed their clarity, cultural suitability, and conceptual equivalence. The content validity was assessed via the Content Validity Index (CVI). The Item-level CVI (I-CVI) was calculated as the proportion of experts rating each item as 3 or 4 on a 4-point relevance scale. The Scale-level CVI was evaluated using the average method (S-CVI/Ave), calculated as the mean of the I-CVI values across all items. The item level CVI (I-CVI) was between 0.80 and 1.00, while scale CVI (S-CVI/Ave) was 0.96 which demonstrates excellent content validity. A pilot test with 10 patients with substance use disorder showed that the items were easy to understand.

Psychometric testing indicated good reliability. Internal consistency was acceptable (Cronbach’s α = 0.924 for temptation; α = 0.709 for confidence). Test–retest reliability of the total self-efficacy score, calculated as confidence minus temptation, was assessed over a two-week interval with the same participants and demonstrated good stability (*r* = 0.878).

Composite scores could range from − 80 to + 80, with higher positive values showing stronger self-efficacy. For description only, scores were grouped into four levels: very low (− 80 to − 40), low (− 39 to 0), moderate (+ 1 to + 39), and high (+ 40 to + 80). A figure was included to show the change in these levels before and after the intervention. These cut-off points were created only for this study and are not part of the original AASE validation.

### Statistical analysis

The collected data were checked for accuracy, coded, and entered SPSS software (version 27) for analysis. Descriptive statistics were used to summarize demographic characteristics, with categorical variables reported as frequencies and percentages and continuous variables as means and standard deviations. Normality of the difference scores was assessed using the Shapiro–Wilk test, which showed non-significant results (*p* = 0.085), confirming that the data met the assumptions of normal distribution. Accordingly, paired-samples t-tests were applied to compare pre- and post-intervention scores within the same group. A significance level of α = 0.05 was set for all statistical analyses. A significance level of α = 0.05 was set for all statistical analyses. In addition, effect sizes were calculated using Cohen’s *d*, computed from paired *t* values (*d* = |*t*|/√*n*), where *t* is the paired-samples *t* value and *n* is the number of participants. This was used to quantify the magnitude of the intervention effect, with 0.2 considered small, 0.5 medium, and 0.8 large [14].

### Data collection procedure

#### Stage 1: Pre-intervention data collection

In the first stage, prior to data collection, the researcher obtained ethical approval from the hospital and secured informed written consent from eligible patients in coordination with the head nurse. Data were gathered through private questionnaire-based interviews while ensuring confidentiality. Each interview lasted 20–30 min, and all completed forms were collected for subsequent statistical analysis.

#### Stage 2: intervention (Life-Skills training Program)

The second stage involved implementing the life-skills training program. The program comprised both theoretical and practical sessions, each lasting approximately 60 min and utilizing a range of teaching methods and materials such as open discussions, brainstorming, role-play, videos, and handouts. The intervention was delivered twice weekly over a period of three months, from March to May 2024. Sessions were incorporated into the ward’s daily schedule to encourage adherence, and learning was reinforced through assignments following theoretical sessions and role-play or simulations after practical ones.

### Program content

The program was developed by the researcher, reviewed by academic supervisors, and grounded in previous literature (e.g., [18, 32, 36, 46, 55]). A total of 15 sessions were delivered, consisting of five theoretical and ten practical sessions. The theoretical component provided patients with essential knowledge about substance use disorders (SUDs), including their classifications, risk factors, impacts on psychological and social functioning, misconceptions, stages of recovery, relapse concepts, and the role of self-efficacy. The practical component focused on skill acquisition to strengthen coping and relapse prevention, covering problem-solving, positive thinking, time management, refusal and assertiveness skills, negotiation, non-violent communication, anger management, relaxation techniques, meditation, and emotional regulation. Each session started with a review of homework and ended with discussion and clarification of questions to ensure understanding. A final closure session was dedicated to program evaluation.

**Table:** 

no.	Session	Objectives
1.	Introductory session	Identify the program purpose, session, and schedule
2.	Overview of Substances Use Disorders	Recognize the Concepts associated with Substances Use Disorders
3.	Impact of Substances Use Disorders	Recognize the impact of Substances Use Disorders on an individual’s life skills and self-efficacy
4.	Recovery stages	Recognize Stages of recovery and recovery from addiction (characteristics and needs of each stage)
5.	Relapse	Illustrate Stages of relapse that may occur widely during recovery
6.	Problem-Solving Skills	Perform problem-solving skills in real situations
7.	Positive Thinking	Perform positive thinking
8.	Time Management	Apply daily Time Management
9.	Refusal Skills	Apply the refusal Skills in everyday life situations (high risk situations)
10.	Assertiveness skill	Demonstrate assertive behaviors in actual situations.
11.	Negotiation Skills	Perform Negotiation Skills
12.	Non-Violence Communication techniques	Apply empathy as a principle of change and NVC in real situation
13.	Anger Management techniques	Manage anger situations
14.	Meditation techniques	Apply Meditation techniques daily
15.	Emotional Regulation techniques	Demonstrate emotional regulation techniques with any life situations
16.	Closure Session	Evaluate life skills training program.

### Program implementation and attendance monitoring

To ensure program fidelity and attendance, the researcher followed a structured session guide that had been validated by supervisors. Attendance was reinforced through reminders from ward staff, and the integration of sessions into the daily routine promoted regular participation. Of the 45 participants who enrolled, 42 completed the entire program, reflecting a low dropout rate of 6.7%. Patients who missed sessions received a brief recap to maintain continuity. All sessions were delivered by the primary researcher, who also provided continuous feedback to enhance patient motivation and commitment.

#### Stage 3: Post-intervention data collection

In the third stage, post-intervention data collection was conducted. Using the same confidential questionnaire format. Completed forms were subsequently prepared for statistical analysis, and pre- and post-intervention results were compared to evaluate the effectiveness of the life-skills training program.

## Results

Table (1) shows that the majority of participants were young adults aged 20–29 years (53.3%; M = 29.84, SD = 6.05). Most were single (71.1%), and more than half (60%) had secondary education or less. Regarding occupation, 44.4% were unemployed and 40.0% engaged in manual work. Most resided in urban areas (86.7%). Financial strain was common, with 62.2% reporting insufficient income. A majority (64.4%) lived in families of four or more members.

**Table 1:** Distribution of socio-demographic characteristics of the patients with substances use disorders (n=45)

Socio-demographic characteristics	n	%
Age (years)		
20 to 29	24	53.30
30 to 39	16	35.60
≥ 40	5	11.10
Mean±SD	29.84± 6.04	
Marital status		
Single	32	71.10
Married	9	20.00
Divorced	2	4.40
Widowed	2	4.40
Educational level		
Read/write.	12	26.70
Primary	6	13.30
Secondary	14	31.10
University or more	13	28.90
Occupation		
Don’t work.	20	44.40
Handicraft’s work	18	40.00
Administrative job	7	15.60
Residence		
Urban	39	86.70
Rural	6	13.30
Monthly income		
Enough	4	8.90
Fairly enough	13	28.90
Not enough	28	62.20
Number of Family members		
2	3	6.70
3	13	28.90
≥ 4	29	64.40

Tables (2) presents a comprehensive overview of the addiction history of the studied patients, highlighting the patterns and types of substances abused. The data indicate Polysubstance use was prevalent. Opiates were the most frequently abused, particularly heroin (77.8%). Alcohol use was reported by 48.9%, cannabis by 24.4%, and tramadol by 31.1%. Synthetic drugs such as methamphetamines (“Ice,” 44.4%) and synthetic cannabinoids (e.g., Vodo, 13.3%) were also reported. Tobacco use was universal (100%), and nearly half smoked shisha (46.7%).

**Table 2:** Distribution of abused substances among the patients with substance use disorders (n=45)

Abused Substances	n	%
Opiates*		
Heroin: Diacetylmorphine	35	77.80
Morphine	2	4.40
Codeine	2	4.40
Hypnotic pills*		
Valium	0	0.00
Rohypnol	1	2.20
Alcohol*	22	48.90
Marijuana*		
Banjo (banjo)	7	15.60
Cannabis	11	24.40
Hallucinations pills and Amphetamines*		
Ecstasy	5	11.10
Apetryl	20	44.40
Stimulants*		
Tramadol	14	31.10
Cocaine	0	0.00
Others*		
“Ice” Methamphetamine hydrochloride	20	44.40
“Shabo” Methamphetamine hydrochloride	6	13.30
“Shaar” Mephedrone	7	15.60
“Crystal” Methamphetamine hydrochloride	1	2.20
“Vodo” Synthetic Cannabinoid	6	13.30
“Astrox” Synthetic Cannabinoid Blend	4	8.90
Smoking*		
Cigarettes	45	100.00
Shisha	21	46.70

*The answers aren’t mutually exclusive

Tables (3) illustrates key aspects of the clinical addiction history among the studied patients. Most participants (64.4%) reported an addiction duration of ≥ 10 years. Family history of addiction was present in 51.1%, mainly among first-degree relatives (44.4%). One third (33.3%) had legal problems. About 46.7% had previous voluntary hospitalizations, 11.1% involuntary, while 42.2% were admitted for the first time. Nearly half (48.9%) reported one relapse, while 37.8% had ≥ 3 relapses. The most common reason for not seeking professional help during relapse episodes was complex admission procedures (40.0%), followed by treatment cost (24.4%).

**Table 3:** Distribution of the patients studied with substance use disorders according to their clinical data (addiction history) (n=45)

Clinical data	n	%
Duration of addiction		
Less than 1 year	3	6.70
1 -<5 years	3	6.70
5 -<10 years	10	22.20
≥ 10 years	29	64.40
Family History of Addiction		
Yes	23	51.10
No	22	48.90
Relationship degree with the other addict family member		
1^st^ degree (father & brothers)	20	44.40
2^nd^ degree (uncles & cousins)	3	6.70
Legal Problems due to addiction		
Yes	15	33.30
No	30	66.70
Previous hospitalization for treatment (n=26)		
Previous Voluntary Hospitalized Admission	21	46.70
Previous Involuntary Hospitalized Admission	5	11.10
The number of relapses times		
1	22	48.90
2	6	13.30
≥ 3	17	37.8
Reasons for not seeking professional help during relapse episode		
Unavailability of places for treatment	9	20.00
Expensive cost of treatment	11	24.40
Poor communication with health care team	4	8.90
Complex admission procedure	18	40.00
Easy access to abused substances during Treatment	3	6.70

Table (4) presents current addiction history among patients with substance use disorders. Nearly three quarters were very or highly satisfied with current treatment (73.3%). Craving was frequent, with 53.3% reporting at least one episode in the past 24 hours. Most cravings were short (1–4 hours, 48.9%), but 15.5% lasted over 8 hours. Relapse occurred within one week for 35.6%. After relapse, most resumed the same dose (66.7%).

**Table 4:** Distribution of the studied patients with substance use disorders according to their clinical data (current addiction history) (n=45)

Clinical data (current addiction history)	n	%
Frequency of craving during the last 24 hrs.		
None	5	11.10
1	24	53.30
2	10	22.20
≥3	6	13.30
Duration of craving to substance during the last 24 hours		
Never	5	11.10
Too short (1hr < 4hrs)	22	48.90
Short (4hrs <8hrs)	11	24.40
Long (8hrs <16hrs)	5	11.10
Too long (16hrs < 24 hrs.)	2	4.40
Time between quit addiction and relapse		
Day -week	16	35.60
> week –month	10	22.20
> month –year	6	13.30
> year	13	28.90
First dose taken after relapse		
less than usual	8	17.80
The same dose	30	66.70
More than usual	7	15.60
Satisfaction level with treatment now		
Slightly Satisfied	3	6.70
Moderately Satisfied	9	20.00
Very Satisfied	11	24.40
Highly Satisfied	22	48.90
Slightly Satisfied	3	6.70

**Fig. 2:**
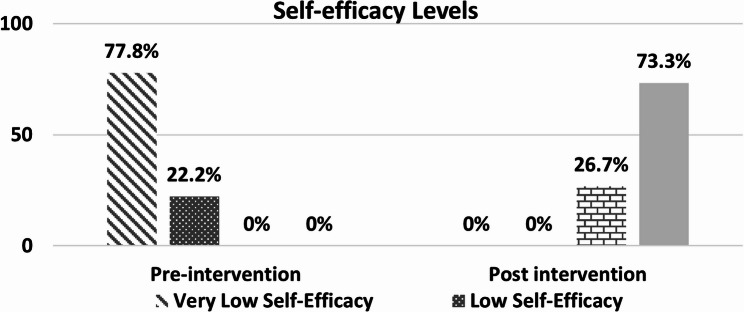
Percentage distribution of self-efficacy levels among the studied patients with substance use disorders pre and post intervention of Life-Skills Training Program (n=45)

Figure (2) displays the percentage distribution of self-efficacy levels among the studied patients with substance use disorders before and after participation in the Life-Skills Training Program. Before the intervention, 77.8% had very low and 22.2% had low self-efficacy. After the program, 73.3% reported high and 26.7% moderate self-efficacy. None remained in the low or very low categories.

Table (5) demonstrates a substantial and statistically significant reduction across all temptation domain subscales following the intervention (p < 0.001). The largest change was observed in the cravings/urge’s situations subscale from mean =21.33±1.638 preintervention to 8.31±1.474 post intervention (d = 6.61). The marked decrease in scores across all subscales and the total temptation score from M = 79.42, SD = 6.77 pre-intervention to M = 32.98, SD = 3.88 post-intervention (d = 6.61).

**Table 5:** Comparison of Pre- and Post-Intervention Scores on Temptation Domain Subscales among Patients with Substance Use Disorders (n = 45)

**Temptation Domain subscales**	**Pre-Intervention **		**Post-Intervention **		*t-***test**	**95% CI ** **(Lower–Upper) **	*p***. value**	*d*
	**Mean**	**SD**	**Mean**	**SD**				
Negative affect	18.82	2.40	8.24	1.09	28.38	[9.83–11.33]	<0.001	4.23
Social/positive	19.40	2.41	8.20	1.34	29.72	[10.44–11.96]	<0.001	4.43
physical and other concerns	19.87	1.72	8.22	1.08	37.75	[10.44–11.96]	<0.001	5.63
Cravings/urges situations	21.33	1.63	8.31	1.47	46.24	[12.46–13.55]	<0.001	6.89
**Total temptation **	79.42	6.76	32.98	3.88	44.37	[44.33–48.55]	<0.001	6.61

*p-*value <0.001 highly significant

df=44

d=Cohen’s conventional benchmarks (0.2≈Small, ، 0.5≈Medium, 0.8≈Large)

Table (6) shows that all subscales of the Confidence Domain (Negative Affect, Social/Positive, Physical and Other Concerns, Cravings and Urges Situations) showed a statistically significant increase from pre- to post-intervention, with p-values <0.001. The largest effect was in physical/other concerns (M = 8.27 → 19.16, d = 5.38). The total confidence scores nearly doubled post intervention from 33.04 ± 4.10 to 77.44 ± 8.35 (d = 5.18). The direction of the t-values is negative because the post-intervention scores were significantly higher, indicating improvement.

**Table 6:** Comparison of Pre- and Post-Intervention Scores on confidence Domain Subscales among Patients with Substance Use Disorders (n = 45)

**Confidence Domain subscales**	**Pre-Intervention **		**Post-Intervention **		*t-***test**	**95% CI ** **(Lower–Upper) **	*p***. value**	*d*
	**Mean **	**SD**	**Mean **	**SD**				
Negative affect	8.18	1.23	19.18	2.24	−31.72	[−11.69 – −10.30]	<0.001	4.73
Social/Positive	8.24	1.38	19.76	3.03	−22.35	[−12.55 – −10.47]	<0.001	3.33
Physical and other concerns	8.27	1.11	19.16	1.99	−36.07	[−11.50 – −10.28]	<0.001	5.38
Cravings/Urges situations	8.36	1.44	19.36	2.09	−29.57	[−11.75 – −10.25]	<0.001	4.41
**Total confidence**	33.04	4.10	77.44	8.35	−34.78	[−46.97 – −41.83]	<0.001	5.18

*p-*value <0.001 highly significant

df= 44

d=Cohen’s conventional benchmarks (0.2≈Small, ، 0.5≈Medium, 0.8≈Large)

Table (7) reveal that total self-efficacy improved substantially (M = –46.38 ± 6.91 → 44.47 ± 8.71, t = –50.27, p < 0.001, d = 7.49), reflecting a strong intervention effect.

**Table 7:** Comparison of Pre- and Post-Intervention Scores of Self Efficacy among Patients with Substance Use Disorders (n = 45)

Total Self Efficacy	Pre-Intervention		Post-Intervention		*t-*test	*95% CI* *(Lower–Upper) *	*p*. value	*d*
	Mean	SD	Mean	SD				
	−46.38	6.91	44.47	8.71	−50.27	[−94.49 – −87.20]	<0.001	7.49

*p-*value <0.001 highly significant

*df*=44

d=Cohen’s conventional benchmarks (0.2≈Small, ، 0.5≈Medium, 0.8≈Large)

## Discussion

This study aimed to evaluate the effect of a life-skills training program on self-efficacy among patients with substance use disorders. The findings showed clear improvements in self-efficacy across all domains—negative affect, social/positive pressure, physical and other concerns, and cravings/urges—after the intervention. These results support the idea that life-skills training can enhance coping abilities, reduce relapse risk, and promote recovery. This is in line with Bandura’s self-efficacy theory, which highlights the role of perceived confidence in managing difficult situations.

### Demographic characteristics

More than half of patients were young adults (20–29 years; mean age 29.84 ± 6.04), and more than two thirds were single. Also, most of them were from urban areas with limited education. Additionally, about two thirds of them had low income, about half didn’t work and large family members. These factors suggest economic and social vulnerabilities that may increase the risk of substance use.

The researcher’s sample is largely comprised of economically disadvantaged, single young men in urban areas. These demographic traits are crucial because they’re linked to various risk factors for substance use. Specifically, the text highlights that limited social support (indicated by marital status), lower educational attainment, economic instability (indicated by monthly income), and increased exposure to stressors (from larger family size and urban living) are key factors influencing the onset and persistence of substance use. Understanding these characteristics is essential for creating effective and targeted interventions.

Similar findings were reported by [16, 29], while [13] reported an older mean age. Studies such as [6, 18, 19, 22] showed mixed results regarding marital status, education, occupation, and income. Overall, our results indicate that early adulthood, financial strain, and limited social support are important risk factors for substance use and should be considered when planning interventions.

### Past clinical history

Regarding abuse substances, Opiates, especially heroin, were the most commonly abused substances, followed by alcohol, methamphetamine hydrocholoride “ice” and Apetryl. Tramadol use was also reported, while cocaine use was absent. Considering all patients smoked cigarettes.

This pattern aligns with regional trends in opioid misuse, reflecting the high addictive potency and widespread availability of heroin. In contrast, cocaine was not reported, potentially due to its higher cost or limited accessibility. The emergence of new psychoactive substances presents significant public health concerns given their potency, unpredictable effects, and growing popularity, particularly among younger populations. Additionally, smoking appears as a prevalent habit and likely functions as a gateway behaviour linked to broader substance use. Collectively, the clinical data reveal a complex addiction profile characterized by polysubstance use, elevated rates of opiate and stimulant abuse, and increasing involvement with synthetic psychoactive drugs. These findings highlight the imperative for comprehensive, multidimensional treatment strategies that effectively address both traditional and emerging substance dependencies.

These results are consistent with [2, 4, 28] who found heroin to be the most common drug. Tobacco use was universal, supporting its role as a gateway substance. This pattern reflects regional and global concerns about polysubstance use and the growing problem of new psychoactive substances. In Contrast with findings of [53] who stated more than half addict stimulant, near than half cannabis, more one third heroin while agreed majority poly substances, and [30, 50] who stated Cocaine, cannabis, and painkillers were the most utilized substances.

### Clinical profile

About two thirds patients had been using substances for more than 10 years, more than half had a family history of substance use, mainly among first-degree relatives, and one third had legal problems. More than half had repeated hospitalizations.

The researcher highlights that prolonged substance use reflects chronic dependency resistant to short-term interventions and linked to severe biopsychosocial harms. Family history indicates genetic and environmental influences, while legal consequences reveal significant social and judicial challenges due to impaired judgment and substance illegality. Treatment histories often show a revolving-door pattern, suggesting inadequate aftercare and persistent risk factors. Similar findings were reported by [10, 52].

Nearly half of participants had more than one relapse, and the main barriers to seeking treatment during relapse were complex admission procedures, high cost, and limited availability of services. These findings point to substantial structural and systemic barriers that may undermine sustained recovery and discourage patients from seeking timely help. although these remain critical issues in certain treatment environments.

The [34], presented several policies regulating the operation and services of addiction treatment centers, which are diverse and often difficult for service recipients to understand. Additionally, there may be individual cases where some required official documents are unavailable, alongside variations in fees and medical insurance depending on the categories of service recipients.

### Current clinical history

In the current study, craving was frequent, usually short but sometimes prolonged, and many patients relapsed within the first month of abstinence with the same dose or higher than previous. Additionally, around half of patients were satisfied with current treatment.

The variations in tolerance, perceived need, or self-regulation attempts may explain relapse patterns, but resuming the same or higher doses after abstinence raises overdose risk due to reduced tolerance. This underscores the necessity of addressing structural barriers, managing cravings, and implementing intensive relapse-prevention especially in early abstinence. The findings emphasize the critical need for strong post-discharge support and follow-up within the initial weeks after treatment to improve recovery outcomes.

The results were in the same [35] craving were higher for individuals with high substance-dependence levels as compared to ones with low substance-dependence levels. Also [39], merge maladaptive changes due to craving, sustaining drug intake and promoting relapses. Moreover [54], show craving severity at week 12 with two drugs. These findings emphasize the importance of post-discharge follow-up and relapse-prevention strategies, especially in the early stages of recovery.

### Effect of life-skills training on self-efficacy

The intervention led to notable reductions in temptation and increases in confidence across all domains. Patients reported better emotional regulation, resistance to social pressure, and control over cravings.

The largest change was observed in the cravings and urges situations, indicating that the intervention was particularly effective in helping participants resist situational triggers for substance use. The marked decrease in scores across all subscales and the total temptation reflects a considerable improvement in participants’ perceived ability to manage temptation. Psychologically, the decline in negative affect and social/positive temptation subscales indicates an enhanced ability to manage emotional distress and social cues without resorting to substance use.

From a clinical perspective, the significant drop in physical and other concerns and cravings and urges situations subscales highlights improved behavioural control and reduced physiological reactivity to triggers commonly associated with relapse. This shift reflects strengthened self-efficacy and cognitive-behavioural resilience, both of which are critical in maintaining abstinence and supporting long-term recovery.

These findings strongly suggest that the intervention was effective in enhancing the self-confidence of patients in resisting substance use across a variety of high-risk scenarios. The increase in confidence scores across subscales indicates: Improved emotional regulation (Negative Affect subscale), Greater social assertiveness and resistance to peer influence (Social/Positive), Enhanced ability to cope with physical discomfort and stress-related triggers (Physical and Other Concerns), and stronger control over cravings and urges, which are core challenges in relapse prevention.

Clinically, this change in confidence scores reflects a positive therapeutic response, likely underpinned by Positive thinking, behavioural rehearsal, and reinforcement of coping strategies during the intervention. The notable elevation in total Confidence Score reflects an improvement in self-efficacy, a critical protective factor against relapse and a key predictor of sustained abstinence in substance use disorder recovery.

These results are consistent with [10, 12, 24–26], who also reported that life-skills training improves resilience, problem-solving, and social functioning. Higher self-efficacy is known to predict longer abstinence and lower relapse, as confirmed by [11, 31, 40, 63].

### Implications for practice

Our findings suggest that life-skills training is an effective and low-cost psychosocial intervention. It can improve self-efficacy, reduce relapse risk, and support rehabilitation. Integrating structured life-skills programs into treatment plans may be especially useful for young, economically disadvantaged patients.

### Strengths and limitations

The study used a validated, adapted tool with good reliability and showed large effect sizes. However, this study has several limitations that should be acknowledged as.

First, the absence of a control group limits the ability to establish causal inferences regarding the effect of the life-skills training program. To partially address this, the sample size was increased beyond the minimum requirement to enhance statistical power and reliability of the findings. Future research employing randomized controlled or longitudinal designs is recommended to confirm these results and improve generalizability.

Second, content validity was assessed using the Content Validity Index (CVI), which confirmed clarity and cultural appropriateness of the adapted tool. However, confirmatory factor analysis and calculation of content validity indices (e.g., CVI with larger panels, modified kappa) was not conducted due to the limited sample size. Future studies with larger and more diverse samples are recommended to examine the factor structure and measurement invariance of the instrument.

Third, no psychiatrist participated in the data collection. Therefore, diagnoses were verified through official medical records documented by qualified psychiatrists.

The study sample consisted of male patients due to administrative and logistical constraints rather than intentional selection bias. While this may limit the generalizability of the findings to female populations, it does not affect the internal validity of the study. Future studies are encouraged to include both genders and multiple treatment facilities to allow for gender-based comparisons.

## Conclusion

This study concluded that: majority of patients use poly substances, many risk factors maybe led to substances use disorders and increase relapse rate such as young adult stage, single life, low education level, unemployment, and over family member that contributed to levels self-efficacy before intervention than post intervention. Life-skills training program had improvement self-efficacy among patients with substance-use disorders.

## Supplementary Information

Supplementary Material 1

Supplementary Material 2

Supplementary Material 3

Supplementary Material 4

## Data Availability

The data presented in this study are available on request from the corresponding author.

## Abbreviations

SUDs: Substance Use Disorders
GSMHAT: General Secretariat of Mental Health and Addiction Treatment
AASE: Alcohol Abstinence Self-Efficacy Scale
DSM: Diagnostic and Statistical Manual of Mental Disorders
WHO: World Health Organization
UNODC: United Nations Office on Drugs and Crime
SAMHSA: Substance Abuse and Mental Health Services Administration
ASE: Abstinence Self-Efficacy
