# Supplementary material for: Life-skills training program: its effect on self-efficacy among patients with substance use disorders
Source: BMC Psychol. 2026 Jan 21;14:119. doi: 10.1186/s40359-025-03907-2 (PMC12849481; doi:10.1186/s40359-025-03907-2)
Supplement: Supplementary file 1 — Supplementary Material 1 [file 40359_2025_3907_MOESM1_ESM.pdf]

{Module Name} Module

Alcohol Abstinence Self-Efficacy Scale

Agency Name: ________________________ **Site Name:** ________________________

ID #: ________________________ **Date:** __ __ / __ __ / __ __ __ __

Listed below are a number of situations that lead some people to drink. We would like to know **how TEMPTED you may be to drink in each situation.** Check the answer that best describes the feelings of temptation in each situation at the present time.

	TEMPTED				
**SITUATION**	**Not at all**	**Not very**	**Moderately**	**Very**	**Extremely**
**1.** When I am in agony because of stopping or withdrawing from alcohol use	❑0	❑1	❑2	❑3	❑4
**2.** When I have a headache	❑0	❑1	❑2	❑3	❑4
**3.** When I am feeling depressed	❑0	❑1	❑2	❑3	❑4
**4.** When I am on vacation and want to relax	❑0	❑1	❑2	❑3	❑4
**5.** When I am concerned about someone	❑0	❑1	❑2	❑3	❑4
**6.** When I am very worried	❑0	❑1	❑2	❑3	❑4
**7.** When I have the urge to try just one drink to see what happens	❑0	❑1	❑2	❑3	❑4
**8.** When I am being offered a drink in a social situation	❑0	❑1	❑2	❑3	❑4
**9.** When I dream about taking a drink	❑0	❑1	❑2	❑3	❑4
**10.** When I want to test my willpower over drinking	❑0	❑1	❑2	❑3	❑4
**11.** When I am feeling a physical need or craving for alcohol	❑0	❑1	❑2	❑3	❑4
**12.** When I am physically tired	❑0	❑1	❑2	❑3	❑4
**13.** When I am experiencing some physical pain or injury	❑0	❑1	❑2	❑3	❑4
**14.** When I feel like blowing up because of frustration	❑0	❑1	❑2	❑3	❑4
**15.** When I see others drinking at a bar or at a party	❑0	❑1	❑2	❑3	❑4
**16.** When I sense everything is going wrong for me	❑0	❑1	❑2	❑3	❑4
**17.** When people I used to drink with encourage me to drink	❑0	❑1	❑2	❑3	❑4
**18.** When I am feeling angry inside	❑0	❑1	❑2	❑3	❑4
**19.** When I experience an urge or impulse to take a drink that catches me unprepared	❑0	❑1	❑2	❑3	❑4
**20.** When I am excited or celebrating with others	❑0	❑1	❑2	❑3	❑4

ID #: ________________________ Date: ___ ___ / ___ ___ / ___ ___ ___ ___

Listed below are a number of situations that lead some people to drink. We would like to know **how CONFIDENT are you that you WOULD NOT drink in each situation.** Check the answer that best describes the feelings of confidence in each situation at the present time.

SITUATION	CONFIDENCE				
	**Not at all**	**Not very**	**Moderately**	**Very**	**Extremely**
**21.** When I am in agony because of stopping or withdrawing from alcohol use	❑0	❑1	❑2	❑3	❑4
**22.** When I have a headache	❑0	❑1	❑2	❑3	❑4
**23.** When I am feeling depressed	❑0	❑1	❑2	❑3	❑4
**24.** When I am on vacation and want to relax	❑0	❑1	❑2	❑3	❑4
**25.** When I am concerned about someone	❑0	❑1	❑2	❑3	❑4
**26.** When I am very worried	❑0	❑1	❑2	❑3	❑4
**27.** When I have the urge to try just one drink to see what happens	❑0	❑1	❑2	❑3	❑4
**28.** When I am being offered a drink in a social situation	❑0	❑1	❑2	❑3	❑4
**29.** When I dream about taking a drink	❑0	❑1	❑2	❑3	❑4
**30.** When I want to test my willpower over drinking	❑0	❑1	❑2	❑3	❑4
**31.** When I am feeling a physical need or craving for alcohol	❑0	❑1	❑2	❑3	❑4
**32.** When I am physically tired	❑0	❑1	❑2	❑3	❑4
**33.** When I am experiencing some physical pain or injury	❑0	❑1	❑2	❑3	❑4
**34.** When I feel like blowing up because of frustration	❑0	❑1	❑2	❑3	❑4
**35.** When I see others drinking at a bar or at a party	❑0	❑1	❑2	❑3	❑4
**36.** When I sense everything is going wrong for me	❑0	❑1	❑2	❑3	❑4
**37.** When people I used to drink with encourage me to drink	❑0	❑1	❑2	❑3	❑4
**38.** When I am feeling angry inside	❑0	❑1	❑2	❑3	❑4
**39.** When I experience an urge or impulse to take a drink that catches me unprepared	❑0	❑1	❑2	❑3	❑4
**40.** When I am excited or celebrating with others	❑0	❑1	❑2	❑3	❑4

Reference: DiClemente CC; Carbonari JP; Montgomery RPG; Hughes SO. The Alcohol Abstinence Self-Efficacy Scale. Journal of Studies on Alcohol 1994;55:141-148.

ID #: ________________________   Date: ___ ___ / ___ ___ / ___ ___ ___ ___

ALCOHOL ABSTINENCE SELF-EFFICACY (AASE)

NEGATIVE AFFECT

18 or 38. When I am feeling angry inside
16 or 36. When I sense everything is going wrong for me
3 or 23. When I am feeling depressed
14 or 34. When I feel like blowing up because of frustration
6 or 26. When I am very worried

SOCIAL/POSITIVE

15 or 35. When I see others drinking at a bar or at a party
20 or 40. When I am excited or celebrating with others
4 or 24. When I am on vacation and want to relax
17 or 37. When people I used to drink with encourage me to drink
8 or 28. When I am being offered a drink in a social situation

PHYSICAL AND OTHER CONCERNS

2 or 22. When I have a headache
12 or 32. When I am physically tired
5 or 25. When I am concerned about someone
13 or 33. When I am experiencing some physical pain or injury
9 or 29. When I dream about taking a drink

CRAVING AND URGES

1 or 21. When I am in agony because of stopping or withdrawing from alcohol use
7 or 27. When I have the urge to try just one drink to see what happens
11 or 31. When I am feeling a physical need or craving for alcohol
10 or 30. When I want to test my willpower over drinking
19 or 39. When I experience an urge or impulse to take a drink that catches me unprepared
