# Supplementary material for: Life-skills training program: its effect on self-efficacy among patients with substance use disorders
Source: BMC Psychol. 2026 Jan 21;14:119. doi: 10.1186/s40359-025-03907-2 (PMC12849481; doi:10.1186/s40359-025-03907-2)
Supplement: Supplementary file 2 — Supplementary Material 2 [file 40359_2025_3907_MOESM2_ESM.pdf]

NIH Public Access
Author Manuscript
J Soc Work Pract Addict. Author manuscript; available in PMC 2013 April 02.

Published in final edited form as:
J Soc Work Pract Addict. 2011 ; 11(3): 245–253. doi:10.1080/1533256X.2011.593445.

Development of a Brief Abstinence Self-Efficacy Measure

PATRICK McKIERNAN, PhD [Adjunct Faculty],
Kent School of Social Work, University of Louisville; and Director, Sober Solutions House, Louisville, Kentucky, USA

RICHARD CLOUD, PhD [Associate Professor],
Kent School of Social Work, University of Louisville, Louisville, Kentucky, USA

DAVID A. PATTERSON [Assistant Professor],
Buffalo School of Social Work, University of New York, Buffalo, New York, USA

SILVER WOLF (ADELV UNEGV WAYA), PhD [Assistant Professor],
Buffalo School of Social Work, University of New York, Buffalo, New York, USA

SEANA GOLDER, PhD [Associate Professor], and
Kent School of Social Work, University of Louisville, Louisville, Kentucky, USA

KARL BESEL, PhD [Associate Professor]
School of Public and Environmental Affairs, Indiana University Kokomo, Kokomo, Indiana, USA

Abstract

This study compared the 40-item Alcohol Abstinence Self-Efficacy (AASE) scale with domains of confidence and temptation to a new 12-item version developed by the authors consisting of the same domains. There were 126 participants who completed the survey. Psychometric analysis demonstrated high reliability and validity consisting of high correlations between domains of confidence (α = .92) and temptation (α = .88) in the 40-item version of the scale compared to the briefer version. The 12-item version appears to provide a clinically reliable and valid measure of AASE domains of confidence and temptation, providing a more efficient tool for clinical practice.

Keywords

abstinence; alcohol treatment; confidence; measurement; scale; self-efficacy; substance abuse treatment; temptation

The last decade has seen a shift in the evaluation of clinical practice. More than ever, social work treatment providers are being confronted with the demands of integrating research findings into practice in the hope of improving the effectiveness of real-world treatment. Administration of a brief, reliable, and valid addiction self-efficacy scale would provide a quick clinical assessment of one of the best predictors of substance abuse treatment outcomes, as self-efficacy has been posited to be an important therapeutic mediating factor according to Prochaska and DiClemente's (1982, 1983) change theory, motivational interviewing (Miller & Rollnick, 2002), and cognitive and behavioral theory (Monti, Kadden, Rohsenow, Cooney, & Abrams, 2002; Morgenstern & McKay, 2002).

Copyright © Taylor & Francis Group, LLC

Address correspondence to Patrick McKiernan, Sober Solutions House, 1124 Logan Street, Louisville, KY 40204, USA. patrick.mckiernan@ky.gov.

NIH-PA Author Manuscript
NIH-PA Author Manuscript
NIH-PA Author Manuscript

NIH-PA Author Manuscript NIH-PA Author Manuscript NIH-PA Author Manuscript

BACKGROUND

Self-efficacy expectations represent an individual's appraisal of one's ability to carry out a behavior (Bandura, 1989). Self-efficacy expectations involve the formulation of judgments about one's competency to perform a task, rather than about the expected outcome of future performance. Thus, it is a cognitive process that acts as a mediator between the desired outcome and confidence in one's ability to perform that behavior (Velicer, DiClemente, Rossi, & Prochaska, 1990).

Self-efficacy is malleable, with four main sources combining to influence the level at a point in time: one's own performance experiences, observations of the performance of others, verbal persuasion, and emotional or physiological arousal (Bandura, 1986). Self-efficacy is most influenced by personal performance experiences, as these are based on some level of personal mastery. Anticipatory performance also has a strong influence, particularly in those cases in which an individual has a high sense of efficacy and is able to visualize success scenarios and thereby create a positive guide for performance. Conversely, those who judge themselves as inefficacious tend to undermine their performance by dwelling mainly on what can go wrong. Perceived self-efficacy, in which an individual visualizes himself or herself executing activities skillfully enhances subsequent performance (Bandura, 1989).

Self-Efficacy and Substance Abuse

Measurements of abstinence self-efficacy were first introduced in smoking cessation programs (Baer & Lichtenstein, 1988; Coelho, 1984; McIntyre, Lichtenstein, & Mermelstein, 1983). Studies of the self-efficacy construct within smoking cessation programs have been useful in the investigation of both cessation of and reduction in smoking behaviors. In addition, self-efficacy measures in relapse situations have demonstrated a predictive ability in which the level of perceived competence in each type of situation effectively predicts the situation in which relapse might occur.

Numerous studies support therapeutic interventions based on self-efficacy theory for people who exhibit addictive behaviors and who possess ineffective coping responses, positive expectations about the effects of alcohol, conditioned responses, and poor expectations about their ability to cope with high-risk situations (Chung, Langenbucher, Labouvie, Moos, & Pandina, 2001; Goldbeck, Myatt, & Aitchison, 1997; Lemieux, 1998; Maisto, Connors, & Zywiak, 2000; Rohsenow & Monti, 1999; Sklar & Turner, 1999). Examining self-efficacy within the context of these behaviors has produced various instruments designed to measure aspects of self-efficacy as it relates to substance abuse including the Situational Confidence Questionnaire (Annis, 1982), and the Alcohol Abstinence Self-Efficacy Questionnaire (DiClemente, Carbonari, Montgomery, & Hughes, 1994).

The role of self-efficacy within research receives general support as a reliable factor predicting behavior change (Burlington, Reilly, Moltzne, & Ziff, 1989; Maisto et al., 2000; Fiorentine & Hillhouse, 2002). Furthermore, self-efficacy is held as a requirement for change within the transtheoretical stages of change theory (Prochaska & DiClemente, 1982, 1983) as well as popular contemporary treatment approaches including motivational interviewing (Miller & Rollnick, 2002) and cognitive and behavior skills training theories and practices (Monti et al., 2002). In general, research suggests that those individuals who benefit from treatment generally show an increase in efficacy expectations between admission and discharge (Maisto et al., 2000). Thus it appears to be important for counselors to track the level of self-efficacy of their clients during treatment.

NIH-PA Author Manuscript
NIH-PA Author Manuscript
NIH-PA Author Manuscript

THE ALCOHOL ABSTINENCE SELF-EFFICACY SCALE

The preceding discussion indicates that self-efficacy represents a key construct that treatment providers should target and hope to effect. It is important to develop treatment content impacting self-efficacy, but treatment providers might, at a minimum, want to measure self-efficacy at treatment intake, discharge, and perhaps during any posttreatment interviews.

The Alcohol Abstinence Self-Efficacy scale (AASE; DiClemente et al., 1994) is a 40-item questionnaire designed to assess self-efficacy as it applies to alcohol abstinence. The scale presents participants with four categories of high-risk situations related to alcohol abstinence: (a) negative affect, (b) social interactions and positive states, (c) physical and other concerns, and (d) withdrawal and urges. There are 40 high-risk situations briefly described in the scale. Participants respond how "tempted" or "confident" they would be to drink in each situation on a 5-point Likert scale ranging from 1 (*not at all*) to 5 (*extremely*). A composite self-efficacy scale is created by subtracting the scores from confidence minus temptation. Scores are summed separately for temptation and self-efficacy. Psychometric analysis of this scale demonstrated high internal consistency (Spearman–Brown $r = .95$; DiClemente et al., 1994). The main purpose of this article is to report on item reduction, reliability, and initial evidence of validity of a new brief 12-item version of the AASE.

METHODS

This analysis describes the initial development of the 12-item self-efficacy scale that includes two subscales: six items measuring confidence and six items measuring temptation to use. Item selection was based on prior analysis by DiClemente and colleagues (1994), who suggested four factors that are included in each subscale: negative affect, social pressure, physical pain/illness, and thoughts about using.

Participants

Participants were 126 individuals recruited for the study from the Volunteers of America addiction treatment programs who were receiving some level of alcohol or other drug abuse services. Participants were drawn from five alcohol and drug treatment programs that provide both long-term residential and intensive outpatient care. The participants entering these programs were addicted to alcohol or to other drugs, with a minimum of about 2 weeks free from any substances.

Procedures

Respondents were invited and consented to participate in the study approved by the University of Louisville internal review board. They were provided with copies of the instrument and instructed to answer each of the questions based on their current situation. Participants completed the questionnaire within the first week of treatment along with initial admission paperwork. Once the materials were completed, the questionnaire was submitted to their identified counselor and forwarded to the Intensive Outpatient Program Manager for data entry.

RESULTS

In all, 126 individuals (110 male, 16 female) completed the survey. Table 1 describes the sample. Participants' mean age was 34 years with a range of 19 to 65 years. The sample's racial mix was distributed such that approximately 41% of the sample reported that they were African American, 58% reported being white, and 2% reported that they were Native American or other.

NIH-PA Author Manuscript

NIH-PA Author Manuscript

NIH-PA Author Manuscript

Item Selection Process

Factor analysis and item analysis were both used to evaluate the measurement structure of the 12-item scale. A principle components solution showed the following results: first eigenvalue = 5.776, second eigenvalue = 2.283, and third eigenvalue = .727. Inspection of the scree plot as well as the eigenvalue magnitudes strongly indicated two factors. Using a principle axis solution with the communalities being estimated, two factors were extracted and rotated using an oblique rotation, promax. An oblique rotation reflected our assumption that the two factors (i.e., temptation and confidence) would be correlated.

As shown in Table 2, Factor 1 consisted solely of the six confidence items, with loadings that ranged from .62 to .88. In contrast, the loadings of the six temptation items ranged (absolute value) from .01 to .10 and consisted of both negative and positive signs. Factor 2 consisted solely of six temptation items. These items had loadings ranging from .62 to .82 on this factor. The loadings of the six confidence items ranged (absolute value) from .03 to .16 with some having negative and some having positive signs.

Using the results of the factor analysis, an item analysis was then conducted to assess internal consistency reliability coefficients. The six confidence items yielded a coefficient alpha reliability of .916 with item–total correlations ranging from .685 to .819. The six temptation items yielded a coefficient alpha reliability of .878 with item–total correlations ranging from .600 to .761.

Good concurrent validity was established by correlating the 20-item and 6-item versions of the confidence and temptation subscales as well as the composite self-efficacy measure that is scored by deducting the two sub-scales. Correlations between the temptation scales ($r = .811$), temptation ($r = .792$), and the composite measure of self-efficacy ($r = .835$) administered to the sample ($n = 126$) provide initial evidence of construct validity.

DISCUSSION

The 12-item version of the AASE provides a brief clinical method for measuring self-efficacy domains of confidence and temptation. Moreover, use of the measure provides a single item indicator of the four domains originally identified within the 40-item scale: negative affect, social pressure, physical pain/illness, and thoughts about using.

Reduction in the time and effort required to objectively measure this important construct might increase the value and utilization of this clinical measure. As workers are met with increasingly limited direct clinical time, this new very brief tool saves time for both workers and clients (Patterson, Nochajski, Dulmus, & Maguin, in press). Workers using the original 40-item scale can adopt the 12-item scale with some assurance that domains will remain valid and reliable while reducing the paperwork burden of their clients.

The AASE provides a measure of confidence in abstaining from and temptation to use alcohol within four hypothesized situations. Readings of confidence and temptation provide information vital for developing effective interventions, planning treatment, and evaluating outcomes. As the social work profession increases its emphasis on evidence-based interventions in the treatment of addiction, deepening its knowledge of these specific issues become more valuable.

Although this study provides some important findings, there are limitations. There needs to be continued research regarding the strong negative correlation observed in previous studies between confidence and temptation. Modification of the instrument might affect the strength of this correlation enough to justify needing only one measure, either temptation or

NIH-PA Author Manuscript

NIH-PA Author Manuscript

NIH-PA Author Manuscript

confidence, thus potentially eliminating even more items from the scale. There is also no way to generalize these finding beyond the participants who were enrolled in this study.

References

Annis, HM. Situational Confidence Questionnaire. Toronto, ON, Canada: Addiction Research Foundation; 1982.

Baer, JS.; Lichtenstein, E. Cognitive assessment. In: Donovan, DM.; Marlatt, GA., editors. Assessments of addictive behaviors. New York, NY: Guilford; 1988. p. 189-213.

Bandura A. The explanatory and predicative scope of self-efficacy theory. Journal of Social and Clinical Psychology. 1986; 4:359–373.

Bandura A. Human agency in social cognitive theory. American Psychologist. 1989; 44:1175–1184. [PubMed: 2782727]

Burlington TA, Reilly PM, Moltzne JO, Ziff DC. Self-efficacy and relapse among inpatient drug and alcohol abusers: A predicator of outcome. Journal of Studies on Alcohol. 1989; 50:354–360. [PubMed: 2787877]

Chung T, Langenbucher J, Labouvie E, Moos RH, Pandina RJ. Changes in alcoholic patients' coping responses predict 12-month treatment outcomes. Journal of Consulting and Clinical Psychology. 2001; 69:92–100. [PubMed: 11302282]

Coelho RJ. Self-efficacy and cessation of smoking. Psychological Reports. 1984; 54:309–310. [PubMed: 6718624]

DiClemente CC, Carbonari JP, Montgomery RPG, Hughes SO. The alcohol abstinence self-efficacy scale. Journal of Studies on Alcohol. 1994; 55:141–148. [PubMed: 8189734]

Fiorentine R, Hillhouse MP. Drug treatment and 12-step program participation the additive effects of integrated recovery activities. Journal of Substance Abuse Treatment. 2002; 18:65–74. [PubMed: 10636609]

Goldbeck R, Myatt P, Aitchison T. End of treatment self-efficacy: A predictor of abstinence. Addiction. 1997; 92:313–324. [PubMed: 9219393]

Lemieux CM. Detriments of expectation of treatment efficacy among incarcerated substance abusers. International Journal of Offender Therapy & Comparative Criminology. 1998; 42:233–245.

Maisto SA, Connors GJ, Zywiak WH. Alcohol treatment, changes in coping skills, self-efficacy, and levels of alcohol use and related problems 1 year following treatment initiation. Psychology of Addictive Behaviors. 2000; 14:257–266. [PubMed: 10998951]

McIntyre KO, Lichtenstein E, Mermelstein RJ. Self-efficacy and relapse in smoking cessation: A replication and extension. Journal of Consulting and Clinical Psychology. 1983; 51:632–633. [PubMed: 6619375]

Miller, WR.; Rollnick, S. Motivational interviewing: Preparing people to change addictive behavior. 2. New York, NY: Guilford; 2002.

Monti, PM.; Kadden, RM.; Rohsenow, DJ.; Cooney, NL.; Abrams, DB. Treating alcohol dependence: A coping skills training guide. 2. New York, NY: Guilford; 2002.

Morgenstern J, McKay JR. Rethinking the paradigms that inform behavioral treatment research for substance use disorders. Addiction. 2002; 102:1377–1389. [PubMed: 17610541]

Patterson DA, Nochajski TH, Dulmus CN, Maguin G. Organizational resource investments: Do funding policies impact client outcomes? Counselor. (in press).

Prochaska JO, DiClemente CC. Transtheoretical therapy: Toward a more integrative model of change. Psychotherapy: Theory, Research, and Practice. 1982; 19:276–287.

Prochaska JO, DiClemente CC. Stages and processes of self-change of smoking: Toward an integrative model of change. Journal of Consulting and Clinical Psychology. 1983; 51:390–395. [PubMed: 6863699]

Rohsenow DJ, Monti PM. Does urge to drink predict relapse after treatment? Alcohol Research & Health. 1999; 23:225–232. [PubMed: 10890818]

Sklar SM, Turner NE. A brief measure for the assessment of coping self-efficacy among alcohol and other drug users. Addiction. 1999; 94:723–729. [PubMed: 10563037]

Velicer WF, DiClemente CC, Rossi JS, Prochaska JO. Relapse situations and self-efficacy: An integrated model. Addictive Behaviors. 1990; 15:271–283. [PubMed: 2378287]

NIH-PA Author Manuscript
NIH-PA Author Manuscript
NIH-PA Author Manuscript

NIH-PA Author Manuscript
NIH-PA Author Manuscript
NIH-PA Author Manuscript

TABLE 1

Sample Key Characteristics

Variable	
Age	
M	34.4
SD	9.8
Range	19–65

	n	**%**
Gender		
Male	110	87.3
Female	16	12.7
Race		
African American	51	40.5
White	73	57.9
Native American	1	0.8
Other	1	0.8
Program		
Women with children	5	4.0
Women with children	4	3.2
Men’s criminal justice	64	50.8
Men and women intensive outpatient program	13	10.3
Dual diagnosis	14	11.1
Men’s program	25	19.8

Note. N = 126.

NIH-PA Author Manuscript

NIH-PA Author Manuscript

NIH-PA Author Manuscript

TABLE 2

Factor Pattern Matrix

	Factor	
	**1**	**2**
1. How tempted would you be to drink or use drugs when you are emotionally upset (feeling down, angry, afraid, or guilty)?	.088	.783
2. How tempted would you be to drink or use drugs when around or seeing others who are using—such as during celebrations or on vacation?	−.103	.763
3. How tempted would you be to drink or use drugs when you experience physical pain, such as a headache, injury, or are physically tired?	−.056	.619
4. How tempted would you be to drink or use drugs when you have thoughts of using—while either awake or dreaming?	−.029	.812
5. How tempted would you be to drink or use drugs when you are feeling a physical need or craving for drugs or alcohol?	.074	.822
6. How tempted would you be to drink or use drugs when you have an urge to try just one drink or use drugs just once to see what happens?	−.005	.679
7. How confident would you be *not* to drink or use drugs when you are emotionally upset (feeling down, angry, afraid, or guilty)?	.817	−.028
8. How confident would you be *not* to drink or use drugs when around or seeing others who are using—such as during celebrations or on vacation?	.800	−.092
9. How confident would you be *not* to drink or use drugs when you experience physical pain, such as a headache, injury, or are physically tired?	.766	.045
10. How confident would you be *not* to drink or use drugs when you have thoughts of using—while either awake or dreaming?	.872	.051
11. How confident would you be *not* to drink or use drugs when you are feeling a physical need or craving for drugs or alcohol?	.620	−.164
12. How confident would you be *not* to drink or use drugs when you have an urge to try just one drink or use drugs just once to see what happens?	.882	.111
