# Supplementary material for: Life-skills training program: its effect on self-efficacy among patients with substance use disorders
Source: BMC Psychol. 2026 Jan 21;14:119. doi: 10.1186/s40359-025-03907-2 (PMC12849481; doi:10.1186/s40359-025-03907-2)
Supplement: Supplementary file 3 — Supplementary Material 3 [file 40359_2025_3907_MOESM3_ESM.pdf]

The Alcohol Abstinence Self-Efficacy Scale

CARLO C. DiCLEMENTE, PH.D., JOSEPH P. CARBONARI, ED.D., ROSARIO P.G. MONTGOMERY, M.A., AND SHERYL O. HUGHES, PH.D.

Department of Psychology, University of Houston, Houston, Texas 77204-5341

ABSTRACT. This study describes the development and the initial psychometric properties of a 20-item, self-report measure to assess Bandura's construct of self-efficacy applied to alcohol abstinence. Efficacy expectations are theorized to mediate behavior change and moderate effort and effective action. The sample was 174 male and 92 female subjects who came to an outpatient alcoholism treatment clinic. Abstinence efficacy was assessed with subject ratings on a 5-point Likert scale of confidence to abstain from alcohol across 20 different high-risk situations. A parallel set of items assessed subjects' temptation to drink in each situation. This Alcohol Abstinence Self-Efficacy scale (AASE) demonstrated a solid subscale structure and strong indices of reliability and validity. The four 5-item subscales measured types of relapse precipitants labeled negative affect, social positive, physical and other concerns, and withdrawal and urges. Both in structure and mean scores the AASE demonstrated no substantive gender differences. The AASE represents a brief, easily usable and psychometrically sound measure of an individual's self-efficacy to abstain from drinking. (*J. Stud. Alcohol* **55:** 141-148, 1994)

IN HIS social cognitive theory, Bandura (1989) points out that individuals' personal evaluations about their capability to exercise control over events or to perform particular behaviors are a central mechanism of personal agency. Self-efficacy evaluations are hypothesized to mediate all behavior change by influencing motivation, information processing, effort and effective action (Bandura, 1977, 1986). Efficacy beliefs are the product of complex cognitive processing of different sources of efficacy information including performance mastery experiences, vicarious experiences, verbal persuasion and physiological states or arousal (Bandura, 1977, 1989). Thus self-efficacy is a dynamic construct that is being constantly reappraised in light of new information or experiences related to the particular focus of the efficacy evaluation. In addition, efficacy evaluations exhibit a gradient of strength and can be assessed across situations and time in order to understand and predict future actions (Bandura, 1989).

The properties of the self-efficacy construct as described by Bandura are particularly intriguing to addictive behavior researchers (DiClemente, 1986). Treatment often utilizes performance and vicarious experiences as well as verbal persuasion to promote cessation and maintained sobriety. These are considered by Bandura to be important sources for developing efficacy evaluations. Moreover, individuals who are addicted and actually using are particularly inefficacious when confronted by their drug of choice. Individuals in recovery report quite different experiences with respect to their confidence or efficacy to abstain from alcohol or drugs. Some appear too confident or cocky; others believe they must be inefficacious or powerless in order to achieve successful sobriety. Understanding the role of self-efficacy in addictive behavior change can provide valuable information to researcher and clinician.

Self-efficacy has long been considered theoretically relevant for alcoholism treatment and recovery (DiClemente, 1986; Marlatt and Gordon, 1980; Rollnick and Heather, 1982). In their cognitive behavioral model of relapse, Marlatt and Gordon (1985) viewed efficacy evaluations as a critical component of the relapse crisis. Efficacy to cope with high-risk situations protects against relapsing in those situations and first use of the substance. Early research focused on identifying high-risk situations and developing a taxonomy of relapse determinants. Marlatt and Gordon (1985) identified eight basic categories of relapse determinants occurring in intrapersonal and interpersonal situations. Although 16 categories were considered high risk, most reported relapses fell into three categories: (1) negative emotional states, (2) interpersonal conflict and (3) social pressure. The relapse model posits that an individual's ability to cope with high-risk situations increases coping self-efficacy, decreasing the probability of relapse.

Annis and her colleagues (Annis, 1986; Annis and Davis, 1989; Annis and Kelly, 1984) have developed a self-efficacy measure that assesses an individual's efficacy "to resist the urge to drink heavily" in a variety of situations that represent the relapse determinants identified by Marlatt and Gordon (1985). This Situational Confidence Questionnaire (SCQ) has both 100- and 40-item formats and is accompanied by an Inventory of Drinking Situations assessing the frequency of drinking heavily in each situation (Annis and Graham, 1990). Several studies have

Received: July 17, 1992. Revision: February 8, 1993.

examined this efficacy to control heavy drinking. Annis and Davis (1988) found that efficacy expectations increased significantly from intake to a 6-month follow-up among problem drinkers. Similarly, Solomon and Annis (1990) found that efficacy to resist the urge to drink heavily increased significantly during inpatient treatment and intake efficacy was strongly associated with average daily drinking at 3 months following treatment. While the SCQ focuses on drinking heavily as the target behavior and efficacy in the ability to control heavy drinking, a similar scale developed by DiClemente and colleagues (DiClemente et al., 1983) concentrated on an individual's efficacy or confidence to abstain from drinking in a range of situations that were derived from the Marlatt relapse categories and surveys of drinkers in treatment. This scale consisted of 49 items and paralleled cigarette-smoking abstinence self-efficacy scales (DiClemente, 1981; DiClemente et al., 1985; Velicer et al., 1990). The Alcohol Abstinence Self-Efficacy Scale (AASE) assessed both the temptation to drink and the confidence or efficacy to abstain in each situation using subjects' ratings on separate 5-point rating scales.

Using this version of the AASE, DiClemente and Hughes (1990) assessed patients' abstinence self-efficacy in the context of exploring the stages of behavior change in an outpatient alcoholism treatment program. Individuals ($N = 224$) entering treatment were classified into a series of stages of change based on their motivation and preparedness to modify their alcohol consumption behavior. Temptations to drink and confidence (efficacy) to abstain from drinking were assessed across the 49 specific situations of the AASE. Stage-based groups differed significantly on both temptation and efficacy scales with subjects closer to action demonstrating more realistic temptation to efficacy relationships. Discouraged subjects had the highest levels of temptation and lowest levels of efficacy. Using a brief version of a similar scale, another study demonstrated that abstinence self-efficacy differs significantly between groups of long-term sober patients and short-term sober patients with long-time sobriety producing higher efficacy evaluations (Miller et al., 1989).

Self-efficacy evaluations in the addictive behaviors and particularly with alcohol dependence or abuse have proved to be important and interesting predictors and covariates of treatment efficacy (DiClemente, 1986; DiClemente et al., in press). However, several problems exist with the assessment of self-efficacy. One problem area is definitional. What type of efficacy is being assessed? This is particularly true in alcoholism treatment where goals of treatment and type of efficacy should correspond. In this respect efficacy to avoid heavy drinking as defined in the SCQ may fail to capture some important aspects of abstinence efficacy as measured by the AASE. A second area of concern involves the adequacy of the psychometric analyses of the scales. Annis has described the SCQ in some depth. However, the stability of the factor structure has not been adequately established. The initial version of the AASE received support for reliability but was not evaluated extensively. Moreover, male and female differences on these alcohol-related efficacy scales have received scant attention. A third problem concerns the length of the current scales. The long form of the SCQ includes 100 items and the short form has 40. The current version of the AASE has 49 items. Since both of these scales recommend a double administration, one for cue strength assessment and another to evaluate efficacy expectations, the total number of items is doubled. Extensive instruments are cumbersome to use in treatment evaluation studies.

The field of alcoholism research could benefit from a sound, brief, abstinence-oriented efficacy measure that can be used for both men and women who abuse alcohol or are alcohol dependent. This need has been addressed through the modification of the AASE questionnaire. A reduced version of this Alcohol Abstinence Self-Efficacy Scale would be a valuable tool in assessing treatment process and outcome if it is a valid, reliable questionnaire and short enough to be included in evaluation batteries. This study will analyze a brief version of the Alcohol Abstinence Self-Efficacy Scale for its factor structure, relationships with other relevant variables and its utility for use with both male and female populations.

Method

Subjects

Subjects were 266 adults who applied for treatment at the Outpatient Alcoholism Treatment Program at the Texas Research Institute of Mental Sciences over a 24-month period. Subjects all had serious drinking problems but did not currently need medical detoxification nor long-term inpatient treatment as assessed by clinical staff. This sample was 65% male ($n = 174$) and 35% female ($n = 92$) with an average age of 34 years, ranging from 18 to 62. The majority of subjects were white (81.5%) with about 14% black and 4% Hispanic. In terms of current marital status, 32% were never married, 23% were currently married and 43% were separated or divorced. Subjects reported a little over 12 years of education as the mean, with a range from 4 to 21 years. The majority of subjects had at least a high school education.

In order to be admitted to the alcoholism treatment program, patients were required to have a diagnosis of alcohol dependence or abuse. DSM-III diagnoses at intake were available for 225 subjects and demonstrated that 80% of these subjects had received an Axis I diagnosis of alcohol dependence, 16% a diagnosis of alcohol abuse and the remainder various diagnoses of polysubstance abuse or organic substance abuse related status. In addition, ap-

proximately 49.7% of the subjects had Axis II personality disorder diagnoses in addition to their primary diagnoses reflecting the mental health setting of this treatment program.

As assessed by the Alcohol Use Inventory (Wanberg et al., 1977), subjects represented a serious level of alcohol-related problems. They began drinking on average at 16.5 years of age and had been involved in steady problem drinking for an average (±SD) of 9 ± 7.2 years. They had a mean score at the fiftieth percentile on the general alcoholism, alcoholic deterioration and daily quantity of alcohol consumption subscales of the AUI. Comparing samples of subjects on their measures, Wanberg and Horn (1985) found that mean (± SD) percentile score for outpatients (n = 150) on the general alcoholism scale was 44.24 ± 10.6 and was significantly different when compared with first admission inpatients (n = 154) with a mean of 50.37 ± 8.8 and chronic severe inpatients (n = 154) who had a mean percentile score of 54.83 ± 7.6. Compared to these samples, the current study population looked more like first admission inpatients.

Measures

Demographic information sheet. As part of the intake process, all subjects were asked a series of questions that were coded on a standard intake form. These questions focused on age, living situation, education and employment status.

University of Rhode Island Change Assessment Scale (URICA). This scale was developed to measure the stages of change (McConnaughy et al., 1983). It operationally defines four theoretical stages of change (precontemplation, contemplation, action and maintenance) identified by DiClemente and Prochaska (DiClemente and Prochaska, 1982, 1985; Prochaska and DiClemente, 1983, 1984). The scale consists of 32 items, with eight items measuring each of the stage subscales. The items are written so that they are relevant to change of a "problem" that is determined by the subjects. Internal consistency for each subscale was high (coefficient alphas range from .88 to .89). Correlations among the subscales form a simplex pattern consistent with the theory. Responses are given on a 5-point Likert format (1 = strong disagreement to 5 = strong agreement). Subscale scores are summed and scores on each of the four stages are obtained for each subject. For an alcohol-problem population a 28-item version with seven items per subscale was used (DiClemente and Hughes, 1990).

Alcohol Use Inventory (AUI). This 147-item, multiple-choice questionnaire is composed of 22 scales: 16 primary scales that measure the patterns of alcohol use, benefits of drinking and the effects of drinking on personal and interpersonal adjustment (Wanberg et al., 1977); five higher order scales that represent broader dimensions of alcohol use; and a single third-order factor that measures alcoholism in general. Responses are given in a dichotomous presence-absence format or a 3-point response allowing for some gradations. Scores are summed and plotted against a normative sample of over 2,000 hospitalized alcoholics.

Alcohol Abstinence Self-Efficacy Scale (AASE). This scale consists of 49 items representing cues related to drinking. Subjects are requested to respond how "tempted" they would be to drink in each situation on a 5-point scale (not at all = 1 to extremely = 5). Similarly, they are asked to rate how "confident" they are that they would not drink in that situation (abstinence self-efficacy) on a similar 5-point Likert scale (Bandura, 1977, 1989). Scores are summed separately for temptation and self-efficacy. Similar scales developed for smoking and other addictive behaviors have demonstrated relevance and solid psychometric properties (DiClemente, 1986). Initial reliability and validity estimates for this scale demonstrated high internal consistency (Spearman and Brown = .95) and a substantial negative correlation (−.58) between temptation and self-efficacy (DiClemente et al., 1983). In the current study nine items were initially deleted from the scale because they were badly skewed and proved unstable in the preliminary principal components analysis.

Procedure

Clinical care. Subjects were individuals who called or walked in to a community outpatient alcoholism treatment program. If they needed inpatient treatment or emergency care, they were given referrals to other programs. This program offered sliding scale services so individuals without resources frequently came to the program as a last resort and, if the physical symptoms of detoxification were under control, were accepted into the program.

The treatment philosophy of this program relied on a case management system and individualized treatment programming. Group therapy modules included alcohol education, values clarification, alternatives to drinking, socialization and family components, as well as a more traditional insight-oriented group. After intake, individual therapy sessions with the case manager and other treatment staff were available on an as-needed basis. A medication evaluation and follow-up chemotherapy sessions were used to assess and treat withdrawal symptoms and other psychiatric problems. Less than 5% of the subjects received any medication for detoxification on an outpatient basis. Medication evaluations for other psychotropic medication to treat concurrent psychopathology (anxiety, depression, etc.) were given 2 to 6 weeks after detoxification. Very few subjects received medication. Involvement in AA and other relevant self-help support groups was strongly advocated but not made a condition of treatment.

The treatment program was housed in a comprehensive mental health treatment and research facility and referrals to the program came from other clinics as well as directly from the community.

Research. At intake, subjects individually completed the demographic information questionnaire, the URICA, the AUI and the complete AASE questionnaire composed of both the temptation and confidence scales.

Results

Factor analysis and subscale reliability. After we eliminated nine items that did not perform well in early analyses, the 40-item self-efficacy (confidence) scale was factor analyzed using an alpha factor model with oblique rotations to clarify the factor structure. An oblique rotation procedure was used since there was reason to assume that the subfactors of efficacy are correlated. Although seven factors had eigenvalues greater than 1, the data were tested for three, four and five factors since the scree plot indicated that three to five factors were the best fit. Past research indicated that four or five factors could be expected. Using the scree plot and clear factor loading (> .40) on only one factor, a four-factor solution was chosen to best fit the data. The first factor was a large Negative Affect (NA) factor which included items that measured both intrapersonal and interpersonal negative affect. Items from both these potential subscales were highly correlated producing a single first factor. The second factor consisted of items representing social situations as well as using alcohol to enhance positive states and was labeled the Social/Positive (SP) factor. The third factor consisted of varied items representing physical discomfort or pain, concerns about others and dreams about drinking. This factor was labeled Physical and Other Concerns (PO) and represented a combination of several Marlatt and Gordon (1985) relapse categories. The fourth factor was one representing withdrawal, craving and testing willpower and was labeled the Withdrawal and Urges (WU) factor.

The five best items assessed by the highest and clearest (i.e., no loading above .30 on a second factor) factor loading representing each of the factors were then assessed for internal consistency. Table 1 contains the five best loading items which formed each of the four subscales of the AASE and their mean scores. Cronbach alpha values were calculated for each of these subscales. Table 2 contains the alpha coefficients, correlations among the subscales and the overall means and standard deviations for each subscale as well as the total scale.

A factor analysis of temptation responses to the items yielded a similar factor solution. Although there were eight factors with eigenvalues greater than 1, the Scree indicated a four- or five-factor solution was best and the four-factor solution had the clearest unique loadings. All items selected from the efficacy factor analyses to represent the subfactors loaded on the same temptation factors. However, the item loadings were not always as high and there was a bit more overlapping of factor loadings on two factors particularly in the physical and other concerns subscale. Coefficient alpha for the temptation factors were as follows NA = .99, SP = .86, WU = .70 and PO = .60.

AASE subscale relationships. In order to understand the relationships among the subscales of the efficacy scales, four models of possible relationships were tested using a structural equations analysis program and the LISREL 7.16 program (Joreskog and Sorbom, 1979, 1989). The four models tested were the null model (no common factor, four independent factors); the one-factor model (no independent factors, one common factor); a four-correlated-factors model; and a four-factor model with one second-order factor. These models parallel the procedures used to evaluate the smoking abstinence self-efficacy scale (Velicer et al., 1990). A variety of statistics were used to evaluate goodness of fit. The best fitting model was judged to be the four-factor model with one second-order factor.[1] These results are similar to those found with the smoking self-efficacy scale.

TABLE 1. Subscale items and mean (± SD) scores for the alcohol abstinence self-efficacy (AASE) measure

Negative affect	
When I am feeling angry inside.	2.7 ± 1.2
When I sense everything is going wrong for me.	2.4 ± 1.3
When I am feeling depressed.	2.5 ± 1.3
When I feel like blowing up because of frustration.	2.6 ± 1.3
When I am very worried.	2.6 ± 1.2
Social/positive	
When I see others drinking at a bar or at a party.	2.7 ± 1.3
When I am excited or celebrating with others.	2.7 ± 1.3
When I am on vacation and want to relax.	2.7 ± 1.3
When people I used to drink with encourage me to drink.	2.9 ± 1.4
When I am being offered a drink in a social situation.	2.8 ± 1.3
Physical and other concerns	
When I have a headache.	3.6 ± 1.5
When I am physically tired.	3.4 ± 1.3
When I am concerned about someone.	3.3 ± 1.3
When I am experiencing some physical pain or injury.	3.3 ± 1.4
When I dream about taking a drink.	3.6 ± 1.5
Withdrawal and urges	
When I am in agony because of stopping or withdrawing from alcohol use.	3.0 ± 1.5
When I have the urge to try just one drink to see what happens.	2.9 ± 1.4
When I am feeling a physical need or craving for alcohol.	2.7 ± 1.4
When I want to test my willpower over drinking.	3.1 ± 1.4
When I experience an urge or impulse to take a drink that catches me unprepared.	2.8 ± 1.3

Note: Means (± SDs) for each item are given for the 266 subjects in the study.

TABLE 2. Alpha reliability, intercorrelations and means for the subscales of the AASE

Subscales	Alpha	AASE subscales NA	SP	PO	WU	Men (n = 170) Mean (± SD)	Women (n = 88) Mean (± SD)
Negative affect (NA)	.88		.46	.56	.51	13.1 ± 5.1	12.6 ± 5.2
Social/positive (SP)	.82			.41	.44	13.8 ± 4.9	14.3 ± 5.8
Physical and other concerns (PO)	.83				.50	16.9 ± 5.3	17.8 ± 5.3
Withdrawal and urges (WU)	.81					14.5 ± 5.2	15.0 ± 5.1
Total Scale	.92					58.3 ± 16.7	59.7 ± 16.5

Note: Subscale scores represent scores for the five items in each subscale (range 5 to 25) for the 266 subjects. Total scale score represents the sum total of the four subscale scores.

In order to evaluate whether a response factor was responsible for the second-order factor, tests were also made fitting an independent general response factor to the data and then asking if the four-factor with one second-order factor model still fit. Removing the response factor actually improved the goodness-of-fit index for the four-factor with one second-order factor model from .983 to .992. Thus the second-order factor model appears to be content driven rather than a response artifact.

In order to evaluate the factor structure of the temptation to drink scale and the relationship between efficacy and temptation, the same strategy was used. Temptation scores for the same 20 items divided into the four subscales were used to fit the four models described above. Once again the analysis using the LISREL program found a four-factor, single second-order factor solution for the temptation items to best fit the data. Path coefficients for both temptation and efficacy subscales are illustrated in Figure 1.

Relationship between efficacy and temptation. Prior research on similar scales would predict that a moderate negative relationship should exist between the confidence and temptation factors. Therefore, we employed a structural model that allowed for a correlation between the second-order confidence factor and the second-order temptation factor. The correlation between these second-order factors was −.65 and supported the existence of a moderate negative correlation between the two domains. Figure 1 also illustrates the second-order relationship for the self-efficacy (confidence) and temptation scales of the AASE.

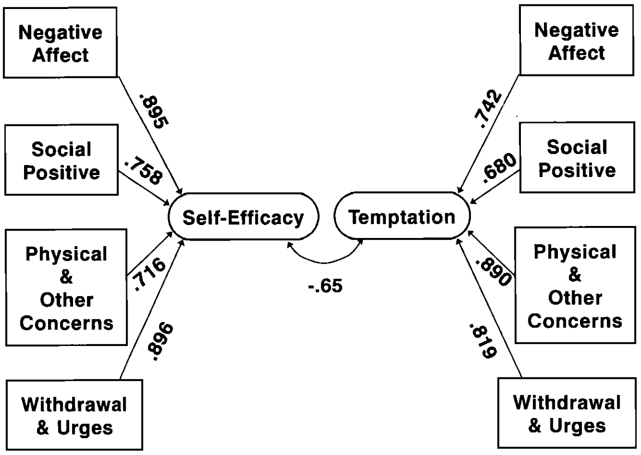

FIGURE 1. Path coefficients of structural model for self-efficacy and temptation factors and relationships

The four efficacy subscales and the four temptation subscales were submitted to a canonical correlation analysis to further test the relationships among them. If the structure of the domains supports more than one confidence factor and more than one temptation factor, a canonical analysis should identify more than one significant canonical variate pair. Four canonical pairs were found to be significantly related (Wilks' lambda, $F = 27.45$, $p < .0001$). This analysis supported the existence of four related subscales as identified in Figure 1.

Construct validity

To examine the validity and independence of the efficacy subscales, subscale scores were correlated with several demographic variables and with the subscales of the Alcohol Use Inventory for the 258 subjects with complete data. Efficacy subscales demonstrated no significant correlations with age and sex of the subjects with one exception: There was a positive relationship between the age of the subjects and the Social/Positive subscale of the AASE ($r = .20$, $p < .01$). Older subjects were more confident in these situations. There were significant but relatively small correlations found between AASE subscales and the AUI primary (Table 3) and secondary (Table 4) subscales. Most relationships were negative indicating that more problems on the AUI related to lower efficacy to abstain from drinking. These relationships are particularly strong for the Negative Affect and the Withdrawal and Urges subscales. The only positive relationship was between use of prior help to stop drinking and the Social/Positive AASE subscale indicating that more prior help and higher abstinence efficacy in social/positive situations shifted together. The efficacy subscale with the least and lowest correlations was the Physical and Other Concerns scale. This subscale demonstrated the greatest independence from alcohol use benefits and consequences.

TABLE 3. First order correlations between AASE and Alcohol Use Inventory subscales

	AASE scales				
AUI primary scales	NA	SP	PO	WU	Total[a]
Social Benefit Drinking	$-.26^{\ddagger}$	$-.29^{\ddagger}$		$-.27^{\ddagger}$	$-.30^{\ddagger}$
Mental Benefit Drinking	$-.17^{\dagger}$	$-.20^{\dagger}$		$-.20^{\dagger}$	$-.21^{\ddagger}$
Gregarious Drinking Style		$-.23^{\ddagger}$			
Obsessive-Compulsive Drinking	$-.16^{\dagger}$			$-.25^{\ddagger}$	$-.20^{\dagger}$
Sustained Drinking Pattern					$-.17^{\dagger}$
Post-Drinking Worry, Guilt, Fear	$-.25^{\ddagger}$	$-.16^{\dagger}$	$-.18^{\dagger}$	$-.32^{\ddagger}$	$-.28^{\ddagger}$
Drinking to Change Mood	$-.36^{\ddagger}$	$-.16^{\dagger}$		$-.24^{\ddagger}$	$-.28^{\ddagger}$
Prior Help to Stop		$.18^{\dagger}$			
Loss of Control When Drinking	$-.28^{\ddagger}$			$-.18^{\dagger}$	$-.21^{\ddagger}$
Social Role Maladaption					
Psychoperceptual Withdrawal	$-.18^{\dagger}$			$-.21^{\ddagger}$	$-.17^{\dagger}$
Psychophysical Withdrawal	$-.23^{\ddagger}$			$-.22^{\ddagger}$	$-.21^{\ddagger}$
Nonalcoholic Drug Use					
Daily Quantity of Alcohol					

$^{\dagger}p < .01$. $^{\ddagger}p < .001$.
[a]Total self-efficacy scores were computed by summing scores from the Negative Affect (NA); Social/Positive (SP); Physical and Other Concerns (PO); and Withdrawal and Urges (WU) subscales.
Note: Only correlations with significance levels ($p < .01$) are included in these analyses. Number of subjects used for these analyses is 258.

TABLE 4. Correlations between AASE and AUI higher order scales

	AASE scales				
AUI higher order scales	NA	SP	PO	WU	Total
Self-Enhancing Drinking	$-.20^{\dagger}$	$-.33$		$-.20^{\dagger}$	$-.27^{\ddagger}$
Obsessive-Sustained Drinking	$-.19^{\dagger}$		$-.16^{\dagger}$	$-.20^{\dagger}$	$-.21^{\ddagger}$
Anxiety Related to Drinking	$-.36^{\ddagger}$	$-.18^{\dagger}$	$-.17^{\dagger}$	$-.32^{\ddagger}$	$-.32^{\ddagger}$
Alcoholic Deterioration	$-.20^{\dagger}$			$-.20^{\dagger}$	$-.19^{\dagger}$
Alcoholic Deterioration[a]	$-.19^{\dagger}$			$-.26^{\ddagger}$	$-.21^{\ddagger}$
General Alcoholism	$-.29^{\ddagger}$	$-.17^{\dagger}$		$-.30^{\ddagger}$	$-.27^{\ddagger}$

$^{\dagger}p < .01$. $^{\ddagger}p < .001$.
[a]Adjunct scale.
Note: Only correlations with significance levels ($p < .01$) are included in these analyses. The number of subjects used for these analyses is 258.

There were certain relationships which supported convergent validity of the subscale constructs. For instance, the Social/Positive subscale was the only one to relate to Gregarious Drinking Style and had the highest correlation with Social Benefit Drinking. Loss of Control When Drinking was most related to the Negative Affect subscale. Withdrawal, General Alcoholism and Alcoholic Deterioration AUI scales were negatively related to both the Negative Affect and Withdrawal and Urges subscales of the AASE. The rather low level of the actual significant correlations (range .16 to .36) appeared to support discriminant validity of the efficacy subscales. In general the AASE subscales correlated appropriately to AUI assessment of the benefits and consequences of drinking to support the independence as well as the relevance of the efficacy construct and the types of relapse categories represented in the subscales.

Relationships between AASE and URICA Stages of Change scales were examined to evaluate overlap between efficacy and motivation. Few significant correlations emerged and those that did represented low to moderate relationships. Scores on the Action subscale of the URICA were consistently and positively related to the AASE subscales (NA = .22, SP = .25, PO = .18, WU = .22) at a significant level ($p < .01$). There were no relationships found with Contemplation and Maintenance subscales and a couple of small but significant relationships with the Precontemplation subscale (NA = .18, SP = .14). AASE subscales appear quite independent of motivation. The relationships between the endorsement of action items and self-efficacy support the relationship of efficacy evaluations with performance accomplishments. This analysis added support for the divergent validity of the AASE subscales and the efficacy construct as measured by the AASE.

Gender differences

A critical question for any scale that measures alcohol-related constructs is whether the scale operates in the same manner for both male and female clients. The goal of this portion of the current research was to search for any gender differences in responses to the confidence and temptation scales and subscales. Before examining for these differences, some basic gender comparisons were made. Male and female subjects were quite similar in terms of alcohol severity. There were no significant differences by gender on most AUI subscales. In particular, there were no differences on the second-order scales of General Alcoholism (male subjects, mean = 31.96 ± 14.3, n = 170; female subjects, mean = 33.65 ± 14.1, n = 88) and the Alcoholic Deterioration subscale (male subjects, mean = 18.2 ± 9.2; female subjects, mean = 17.9 ± 9.1). There was a significant difference on the Self-Enhancing Drinking subscale ($p < .05$) with men demonstrating more self-enhancing drinking (mean = 8.1 ± 3.8) than women (mean = 6.9 ± 3.9). The only other differences indicated that men had more nonalcohol drug use and greater social role maladaptation. The women were significantly higher on Loss of Control when Drinking and Psychophysical Withdrawal subscales. Thus, the samples of men and women appeared comparable with regard to severity of alcohol-related problems and types of drinking. On demographics and drinking history, female subjects tended to be a little older (35 years) and started drinking a little later (18.8 years) than men (33 years and 15.6 years). Thus, efficacy differences, if any, could be attributed to reported abstinence efficacy and not severity differences.

Gender differences on efficacy and temptation scales could occur in multiple ways. There could be (1) differences in the means of temptation and self-efficacy subscales; (2) differences in the variances of these variables; or (3) differences in the covariances between them. The

LISREL program is capable of carrying out a comparative analysis across these three areas simultaneously. Two covariance matrices with mean vectors (men and women) were analyzed to test simultaneously for all three types of differences. A good fit would mean that the structure, variances/covariances and means represented two samples from the same population. This would indicate that men and women did not differ significantly on their responses to the AASE scale. Lack of fit would indicate gender differences. The measures of the goodness of fit for this model indicated a good fit ($\chi^2 = 19.15$, 14 df, $p = .159$; GFI = .96). These fit indices support lack of gender differences across confidence scales and the existence of a common structure, common variances/covariances and common means for male and female subjects. Table 2 illustrates the means and standard deviations for efficacy subscales by gender. There were no differences by gender for temptation subscales.

Discussion

The 20-item Alcohol Abstinence Self-Efficacy (AASE) measure examined in this study demonstrated a solid subscale structure, strong indices of being a reliable and valid measure of abstinence efficacy and no substantial gender differences. The five-item subscales appear to capture major dimensions of the relapse determinant categories as studied by Marlatt and Gordon. Situations that generate Negative Affect, whether intrapersonally or interpersonally derived, appear the most powerful and relevant cues for abstinence efficacy as well as for relapse. Social pressure, social cue and more positive types of situations form a second area of concern. Withdrawal symptoms, urges and testing personal control represent another subset of situations that seem to cluster for efficacy as well as for relapse precipitants. The AASE represents a brief, easily usable, comprehensive and psychometrically sound measure of self-efficacy to abstain from drinking.

Relationships between AASE and AUI subscales support the relevance and independence of the AASE when compared with other constructs measuring alcohol-related problems, patterns and severity. Abstinence efficacy is not simply a reflection of severity of alcohol dependence, withdrawal symptoms or benefits of drinking. Although AASE subscales were significantly correlated with some AUI subscales, the correlations were modest and not all AUI dimensions related to current abstinence efficacy. Some specific AUI constructs like Gregarious Drinking Style and Drinking to Change Mood did correspond to the AASE subscales assessing related situations. The independence of AASE subscales from related motivational constructs was also established through correlations with URICA subscales. These relationships support convergent and divergent validity for the AASE.

The structure of the AASE with subscales that reflect relapse precipitant categories and a second-order factor that supports an overall abstinence self-efficacy parallel the findings of Velicer and colleagues (1990) with smoking abstinence self-efficacy. These scales allow for an examination of abstinence efficacy in specific types of situations that could precipitate relapse as well as an evaluation of an efficacy across all situations. The existence of parallel scales in these two areas of addictive behaviors can enhance the communality of research findings across addictions.

The moderate inverse relationship between temptation and efficacy also parallels findings with smoking abstinence self-efficacy (DiClemente et al., 1985). Temptation appears to be a separate but related construct to efficacy. Temptation scores can be seen as evaluating the cue strength of each situation in terms of its ability to precipitate alcohol consumption. Thus, temptation to drink in one situation can be low and efficacy to abstain quite high. However, this is not always the case particularly during the course of recovery when, despite high temptation, an individual could have moderate to high levels of efficacy to abstain based on skills and commitment. The relationship between efficacy and temptation scores presents a new and fertile field for research. Preliminary indications are that difference scores between temptation and efficacy as well as the correlation between them can provide important information related to stages of behavior change for alcohol dependent clients (DiClemente and Hughes, 1990).

A real contribution of the current study is the inclusion of a significant number of women with similar alcohol-related problem levels and attending the same outpatient program as the men. The substantial number of both male and female subjects enabled us to examine gender differences. Both in structure and mean scores current subjects demonstrated no substantial gender differences. Thus, this scale can be used with both men and women and can be utilized to test for gender by treatment differences. Since the scales are not gender specific, differences found could reasonably be assigned to the gender by treatment effect. This is not to say that differences between some men and women on these scales could not be found or that some additional situations for subgroups of men and women could not provide important information about differences. Items could be added to reflect these special situations or special interests. However, the fact that the AASE is a generic scale that can be utilized with both men and women makes it particularly adapted for alcoholism treatment research.

Further testing of the AASE is currently being carried out with a new sample to address limitations of the current study. One limitation is that the initial factor structure and the model testing were done on the same sample of subjects. The strength of the values found in this study,

however, lead us to believe that a very modest loss of reliability or structure would occur with a new sample. Another limitation is that the sample is an outpatient one. Replication with inpatient and aftercare samples are needed. A third limitation is that the sample is predominantly white. Evaluation of the scale in an ethnically diverse population also would require further research.

In conclusion, the Alcohol Abstinence Self-Efficacy Scale examined in this study provides a vehicle for applying the important social learning construct of self-efficacy to individuals with alcohol abstinence as a goal. Efficacy self-evaluations have already proved valuable in smoking, overeating and anorexia research as well as with individuals having alcohol-related problems. The AASE can help promote research and enhance clinical practice by utilizing efficacy judgments as a means to evaluate current status, treatment outcome, relapse potential and possibly treatment matching. The information provided in the Temptation to Drink scale and efficacy scores on the subscales of the AASE can be valuable to both researchers and clinicians. The solid structure of the AASE supports its use and utility for future research and practice.

Acknowledgments

The collection of these data involved the entire clinical research team from the Alcoholism Treatment Clinic at the Texas Research Institute of Mental Sciences. We are grateful to all who assisted, and we dedicate this article to the memory of Jack R. Gordon, M.D., whose leadership made this research possible.

Note

1. Copies of the tests of the models and goodness-of-fit indices are available from the authors.

References

Annis, H.M. A relapse prevention model for treatment of alcoholics. In: Miller, W.R. and Heather, N. (Eds.) Treating Addictive Behaviors: Processes of Change, New York: Plenum Press, 1986, pp. 407-421.

Annis, H.M. and Davis, C.S. Self-efficacy and the prevention of alcoholic relapse: Initial findings from a treatment trial. In: Baker, T.B. and Cannon, D. (Eds.) Assessment and Treatment of Addictive Disorders, New York: Praeger Pubs., 1988, pp. 88-112.

Annis, H.M. and Davis, C.S. Relapse prevention training: A cognitive-behavioral approach based on self-efficacy theory. J. Chem. Depend. Treat. **2**(No. 2): 81-103, 1989.

Annis, H.M. and Graham, J.M. Situational Confidence Questionnaire, Toronto: Addiction Research Foundation, 1990.

Annis, H.M. and Kelly, P. Analysis of the inventory of drinking situations. Paper presented at the convention of the American Psychological Association, Toronto, 1984.

Bandura, A. Self-efficacy: Toward a unifying theory of behavioral change. Psychol. Rev. **84**: 191-215, 1977.

Bandura, A. Social Foundations of Thought and Action: A Social Cognitive Theory, Englewood Cliffs, N.J.: Prentice-Hall, Inc., 1986.

Bandura, A. Human agency in social cognitive theory. Amer. Psychologist **44**: 1175-1184, 1989.

DiClemente, C.C. Self-efficacy and smoking cessation maintenance: A preliminary report. Cogn. Ther. Res. **5**: 175-187, 1981.

DiClemente, C.C. Self-efficacy and the addictive behaviors. J. Social Clin. Psychol. **4**: 302-315, 1986.

DiClemente, C.C., Fairhurst, S.F. and Piotrowski, N. The role of self-efficacy in the addictive behaviors. In: Maddux, J.E. (Ed.) Self-efficacy, Adaptation and Adjustment: Theory, Research and Application, New York: Plenum Press, in press.

DiClemente, C. C., Prochaska, J.O. and Gibertini, M. Self-efficacy and the stages of self-change of smoking. Cogn. Ther. Res. **9**: 181-200, 1985.

DiClemente, C.C., Gordon, J.R. and Gibertini, M. Self-efficacy and determinants of relapse in alcoholism treatment. Paper presented at the annual convention of the American Psychological Association, Anaheim, California, August, 1983.

DiClemente, C.C. and Hughes, S.O. Stages of change profiles in outpatient alcoholism treatment. J. Subst. Abuse **2**: 217–235, 1990.

DiClemente, C.C. and Prochaska, J.O. Self-change and therapy change of smoking behavior: A comparison of processes of change in cessation and maintenance. Addict. Behav. **7**: 133-142, 1982.

DiClemente, C.C. and Prochaska, J.O. Processes and stages of self-change: Coping and competence in smoking behavior change. In: Shiffman, S. and Wills, T.A. (Eds.) Coping and Substance Use, San Diego, Calif.: Academic Press, Inc., 1985, pp. 319-343.

Joreskog, K.G. and Sorbom, D. Advances in Factor Analysis and Structural Equation Models, Cambridge, Mass.: ABT Books, 1979.

Joreskog, K.G. and Sorbom, D. DOS-LISREL 7.16, Mooresville, Ind.: Scientific Software, Inc., 1989.

McConnaughy, E.A., Prochaska, J.O. and Velicer, W.E. Stages of change in psychotherapy: Measurement and sample profiles. Psychother. Theory Res. Pract. **20**: 368-375, 1983.

Marlatt, G.A. and Gordon, J.R. Determinants of relapse: Implications for the maintenance of behavior change. In: Davidson, P.O. and Davidson, S.M. (Eds.) Behavioral Medicine: Changing Health Lifestyles, New York: Brunner-Mazel, Inc., 1980, pp. 410-452.

Marlatt, G.A. and Gordon, J.R. (Eds.) Relapse Prevention: Maintenance Strategies in the Treatment of Addictive Behaviors, New York: Guilford Press, 1985.

Miller, P.J., Ross, S.M., Emmerson, R.Y. and Todt, E.H. Self-efficacy in alcoholics: Clinical validation of the Situational Confidence Questionnaire. Addict. Behav. **14**: 217-224, 1989.

Prochaska, J.O. and DiClemente, C.C. Stages and processes of self-change of smoking: Toward an integrative model of change. J. Cons. Clin. Psychol. **51**: 390-395, 1983.

Prochaska, J.O. and DiClemente, C.C. Self change processes, self efficacy and decisional balance across five stages of smoking cessation. In: Engstrom, P.F., Anderson, P.N. and Mortenson, L.E. (Eds.) Advance in Cancer Control: Epidemiology and Research, New York: Alan R. Liss, Inc., 1984, pp. 131-140.

Rollnick, S. and Heather, N. The application of Bandura's self-efficacy theory to abstinence-oriented alcoholism treatment. Addict. Behav. **7**: 243-250, 1982.

Solomon, K.E. and Annis, H.M. Outcome and efficacy expectancy in the prediction of post-treatment drinking behaviour. Brit. J. Addict. **85**: 659-665, 1990.

Velicer, W.F., DiClemente, C.C., Rossi, J.S. and Prochaska, J.O. Relapse situations and self-efficacy: An integrative model. Addict. Behav. **15**: 271-283, 1990.

Wanberg, K.W. and Horn, J.L. The Alcohol Use Inventory (AUI): A Guide to the Use of the Paper-Pencil Version, Denver, Colo.: Multivariate Measurement Consultants, 1985.

Wanberg, K.W., Horn, J.L. and Foster, F.M. A differential assessment model for alcoholism: The scales of the Alcohol Use Inventory. J. Stud. Alcohol **38**: 512-543, 1977.
